# Tesofensine, a novel antiobesity drug, silences GABAergic hypothalamic neurons

**DOI:** 10.1371/journal.pone.0300544

**Published:** 2024-04-24

**Authors:** Claudia I. Perez, Jorge Luis-Islas, Axel Lopez, Xarenny Diaz, Omar Molina, Benjamin Arroyo, Mario G. Moreno, Elvi Gil Lievana, Esmeralda Fonseca, Gilberto Castañeda-Hernández, Ranier Gutierrez

**Affiliations:** 1 Departamento de Neurobiología del Desarrollo y Neurofisiología, Instituto de Neurobiología, UNAM, Campus Juriquilla, Querétaro, Mexico; 2 Department of Pharmacology, Laboratory of Neurobiology of Appetite, CINVESTAV, México, México; 3 Princeton Neuroscience Institute, Princeton, NJ, United States of America; 4 Department of Pharmacology, Cinvestav, México, México; 5 Centro de Investigación sobre el Envejecimiento (CIE), Cinvestav sede sur, México, México; University of Nebraska Medical Center College of Medicine, UNITED STATES

## Abstract

Obesity is a major global health epidemic that has adverse effects on both the people affected as well as the cost to society. Several anti-obesity drugs that target GLP-1 receptors have recently come to the market. Here, we describe the effects of tesofensine, a novel anti-obesity drug that acts as a triple monoamine neurotransmitter reuptake inhibitor. Using various techniques, we investigated its effects on weight loss and underlying neuronal mechanisms in mice and rats. These include behavioral tasks, DeepLabCut videotaped analysis, electrophysiological ensemble recordings, optogenetic activation, and chemogenetic silencing of GABAergic neurons in the Lateral Hypothalamus (LH). We found that tesofensine induces a greater weight loss in obese rats than lean rats, while differentially modulating the neuronal ensembles and population activity in LH. In Vgat-ChR2 and Vgat-IRES-cre transgenic mice, we found for the first time that tesofensine inhibited a subset of LH GABAergic neurons, reducing their ability to promote feeding behavior, and chemogenetically silencing them enhanced tesofensine’s food-suppressing effects. Unlike phentermine, a dopaminergic appetite suppressant, tesofensine causes few, if any, head-weaving stereotypy at therapeutic doses. Most importantly, we found that tesofensine prolonged the weight loss induced by 5-HTP, a serotonin precursor, and blocked the body weight rebound that often occurs after weight loss. Behavioral studies on rats with the tastant sucrose indicated that tesofensine’s appetite suppressant effects are independent of taste aversion and do not directly affect the perception of sweetness or palatability of sucrose. In summary, our data provide new insights into the effects of tesofensine on weight loss and the underlying neuronal mechanisms, suggesting that tesofensine may be an effective treatment for obesity and that it may be a valuable adjunct to other appetite suppressants to prevent body weight rebound.

## Introduction

Obesity is a worldwide health problem that has reached epidemic proportions. Although diet and exercise are the primary treatments for obesity, these activities are often supplemented using appetite suppressants. Tesofensine (NS2330) is a triple monoamine re-uptake inhibitor with an affinity for dopamine (DAT), serotonin (SERT), and norepinephrine (NET) transporters. It has been shown to have antiobesity effects in animal and human studies [1]. Tesofensine significantly reduced daily food intake in rats under a 16-day treatment regimen, leading to a significant and sustained decrease in body weight. However, the anorexigenic effect of tesofensine progressed to tolerance, while the weight loss effect did not [2]. Hence, tesofensine is a dual-action drug with anorexigenic and metabolic properties, increasing energy expenditure. More impressively, tesofensine reduces body weight in high-fat-fed rats more effectively than in chow-fed rats [2, 3]. Moreover, it is known that tesofensine activates α1 adrenergic receptors and, to a lesser extent, dopamine D1 receptors [2–4]. It exhibits potent antiobesity effects, yet the underlying cellular mechanisms are still being actively investigated. This study first aims to identify the neuronal correlates of tesofensine-induced weight loss in the Lateral Hypothalamus (LH) in lean and obese rats.

The LH is a brain region that regulates numerous physiological processes involving seeking and feeding behaviors [5]. Lesions in the LH can cause decreased food intake and weight loss, while stimulation can increase food intake and promote obesity [6, 7]. The LH comprises two major neuronal populations, GABAergic and glutamatergic neurons, that play opposing and bidirectional roles in reward and feeding [8–10]. In mice and primates, activation of LH GABA neurons promotes food intake, while silencing them inhibits food intake [11–13]. In contrast, in mice, the activation of LH glutamatergic neurons inhibits food intake, while their inhibition promotes food intake [10]. However, it is currently unknown whether tesofensine targets these neuronal populations.

A second aim of this study, in mice, is to characterize how tesofensine targets LH GABAergic neurons to modulate feeding behavior. A third aim was to compare in lean rats the anti-obesity effects of tesofensine with phentermine, another appetite suppressant that increases dopamine efflux in the nucleus accumbens and also induces head weaving stereotypy [14, 15]. We also investigated the pharmacological interaction between tesofensine and 5-HTP, a serotonin precursor and appetite suppressant, and found that tesofensine delayed weight loss rebound [16–18]. Finally, we investigated whether tesofensine affects the gustatory perception of sweetness, as it is reported to decrease the craving for sweet food [19]. Overall, our study provides insights into the potential use of tesofensine as an effective treatment for obesity.

## Materials and methods

### Drugs and delivery methods

Several drugs used in the study, including 5-HTP (5-Hydroxytryptophan), carbidopa (CB), and tesofensine, were kindly donated by Productos Medix (Mexico). 5-HTP was dissolved in physiological saline (9% NaCl) at 40°C and injected intraperitoneally. CB was suspended in carboxymethyl cellulose (0.5%) and saline (1:4 ratio) and injected intraperitoneally. Tesofensine was dissolved in saline and injected subcutaneously. Clozapine-N-Oxide (CNO) was dissolved in DMSO/saline (0.1/1ml) and administered intraperitoneally.

### Diets

The control diet of standard chow is composed of 3.43 kcal/g of the following 4.5% lipids, 20% proteins, and 6% carbohydrates (PicoLab, 5053). The High Fat Diet is composed of 4.7 kcal/g: 45% lipids, 20% proteins, and 35% carbohydrates (Research Diet, D12451). Body weight and food intake were measured weekly.

### Subjects: Mice

Male and female adult VGAT-ChR2-EYFP mice (RRID: IMSR_JAX:014548, weighing 20–30 g; n = 4) and weaned Vgat-IRES-cre mice (RRID:IMSR_JAX:016962, after 21 postnatal day; n = 27) were studied. Both mouse types were purchased from The Jackson Laboratory (Sacramento, CA, USA). VGAT-ChR2-EYFP mice were housed in individual standard laboratory cages with *ad libitum* access to water and chow diet (PicoLab Rodent Diet 20, St. Louis, MO, USA). Vgat-IRES-cre mice were placed in a bodyweight monitoring protocol (described below). Mice were maintained in standard laboratory conditions of a temperature-controlled (22 ± 1°C) room with a 12:12 h light-dark cycle (7:00–19:00). The Institutional Animal Care and Use Committee (CINVESTAV) approved all procedures.

### Body weight monitoring protocol

Weaned female or male Vgat-IRES-cre mice were separated into groups of 3–5 mice in standard laboratory cages. They were given in their homecages *ad libitum* access to water and either a standard chow diet (PicoLab Rodent Diet 20, St. Louis, MO, USA) or high fat diet (HFD, Research Diet, D12451). Body weights were measured in the evenings once per week for 3 months.

#### Viral constructs

The Cre-inducible adeno-associated virus (AAVs) were purchased from addgene Watertown, MA USA). For ChR2-eYFP mice (AAV5-EfIa-DIO-hChR2(E123T/T159C)-EYFP, #35509, at a titer of 1x10^13^ vector genome/ml (vg/ml), eYFP-vector (AAV5-EfIa-DIO EYFP), #27056, at a titer of 1.0x10^13^ vg/ml, and hM4D(Gi) (AAV8-hSyn-DIO-hM4D(Gi)-mCherry), #44362, at a titer of 1.0x10^13^ vg/ml. Viruses were divided into aliquots and stored at −80°C before their use.

#### Stereotaxic surgery in mice

After 8–10 weeks of the body weight monitoring protocol, Vgat-IRES-cre mice were individually anesthetized with an intraperitoneal injection of ketamine/xylazine (100/8 mg/kg), and the mice were then placed in a stereotaxic apparatus. A midline sagittal scalp incision was made to expose the skull, and two holding screws were inserted into the skull. A microinjection needle (30-gauge) was connected to a 10 μl Hamilton syringe and filled with adeno-associated virus (AAV). Mice were microinjected with AAV (0.5 μl) at a 0.2 μl/min rate. The injector was left in position for 5 additional minutes to allow complete diffusion. For the expression of ChR2, eYFP, or hM4D(Gi), For expressing ChR2, eYFP, or hM4D(Gi) the microinjection was performed unilaterally in the LH (from bregma (mm): AP -1.3, ML ±1.0, and from dura (mm): DV -5.5). In the case of hM4D(Gi), the mouse’s head was sutured, and one month was allowed for recovery and expression.

#### Optogenetic experiments

After AAVs infection and to deliver blue light stimulation, a zirconia ferrule of 1.25 mm diameter with multimode optical fiber (200 μm, Thorlabs) was implanted in the LH (from bregma (mm): AP -1.3, ML ±1.0, and from dura (mm): DV -5.3). Mice were allowed one month to recover and to obtain a stable expression of ChR2 or eYFP.

#### Optrode LH implantation in mice

The head of the mouse was sutured, and they were allowed 3 weeks for recovery and protein expression. Then, one optrode (16 tungsten wires surrounding a central optic fiber) was unilaterally implanted in the LH (from bregma (mm): AP -1.3, ML ±1.0, and from dura: DV -5.5), and a subcutaneous catheter was inserted subcutaneously for tesofensine administration. Mice were allowed one additional week for recovery.

#### Optogenetic stimulation protocol

A 473-nm laser was intensity-modulated by a DPSS system (OEM laser, UT, USA). Laser pulses were synchronized with behavioral events using Med Associates Inc. software and a TTL signal generator (Med Associates Inc., VT, USA). The patch cord’s optical power was 15 mW and measured with an optical power meter (PM20A, Thorlabs, NJ, USA). However, depending on the efficiency of each fiber, we delivered between 10 and 12.6 mW at the fiber optic tip. Unless otherwise mentioned, the laser was turned on for 2 seconds (at 50 Hz) and off for 4 seconds, with a 10-ms pulse width and a duty cycle of 50%.

#### Open-loop task

Mice were injected subcutaneously with tesofensine (2 mg/kg, 6 mg/kg, or saline) and maintained in their homecage for 30 minutes. After that, mice were placed in an operant chamber (Med Associates Inc., VT, USA) with access to 10% sucrose via licking a central sipper port. Mice received a 5-min block of no stimulation (off) followed by the delivery of the optostimulation pattern (on) for 30 minutes (see *Optogenetic stimulation protocol* section). All licking responses were recorded by a contact lickometer (Med Associates Inc., VT, USA).

#### Chemogenetic silencing

Five mice fed a high-fat diet (HFD) with the expression of hM4D(Gi) were placed in their homecage with an automated feeder (FED 3, Open Ephys; Lisbon, Portugal). Each time mice executed a nose poke, the feeder delivered a chocolate pellet (20 mg, #F05301, Bio-Serv, Nueva Jersey, USA). Tesofensine (2 mg/kg, subcutaneously) and Clozapine-N-Oxide (CNO, 3 mg/kg, intraperitoneally), both drugs or vehicle (DMSO or saline) were administered at 18:30 (30 minutes before the dark cycle begins). The vehicle was always administered in the subsequent session to avoid a carryover effect of drugs. Sessions were counterbalanced between subjects.

#### Electrophysiology in mice

Extracellular recordings of LH in freely moving Vgat-IRES-cre mice Multi-unit recordings were performed using a Multichannel Acquisition Processor system (Plexon, Dallas, Texas) and a Med Associates (Fairfax, VT) interphase to record behavioral events simultaneously. Voltage signals were sampled at 40 kHz and digitalized at 12-bit resolution. Action potentials with a signal-to-noise ratio larger than 3:1 were analyzed. They were identified online using a voltage-time threshold window and a three-principal component contour template algorithm [32]. After the experiment, spikes were sorted using Off-line Sorter software (Plexon Inc.).

Two mice were recorded in a baseline period of 5 min, then saline (0.15 ml, 0.9%) or tesofensine (2 mg/kg) was administered via a subcutaneous catheter. Thirty minutes later, mice received optogenetic stimulation in blocks of 5 min Off-5 min followed by a block On (2s on-4s off) for 40 min (eight blocks total). The same mice were recorded in two sessions with tesofensine (mouse name LH01 n = 15 and LH02 n = 16 neurons, total 31 neurons), whereas in two intercalated and contiguous sessions, the same mice received saline injections (mouse LH01 n = 16 and LH02 n = 20, in total 36 neurons).

#### Histology in mice

Mice were anesthetized with sodium pentobarbital (75 mg/kg) and then perfused intracardially with PBS 1x and paraformaldehyde at 4%. Their brains were removed and stored in 4% paraformaldehyde solution for 48-h hours and put in a 30% sucrose solution for 72-h hours. The brain was sliced, and sections of 40 μm were mounted in Dako fluorescence mounting medium. Immunofluorescence was observed using a ZEISS LSM 800 confocal microscope.

### Subjects: Rats

Adult male Wistar rats were used in all experiments: From weaning, thirty-three rats were housed in pairs until they were 12 weeks of age, after which they were housed individually. During those 12 weeks, the rats were given either a high-fat diet (HFD) or a chow diet and had *a*d libitum access to water. Six male rats (n = 3 with HFD, n = 3 with chow food) had electrodes implanted in their LH for electrophysiological recordings.

A total of 24 male rats, 330–370 g, were used for the locomotion and stereotypy experiments. The rats were housed in their home cages in pairs and had *ad libitum* access to food and water. In the acrylic box, rats were given one pellet of chow and one high-fat diet (HFD) attached to a side wall. In the isobologram studies, lean rats (n = 142) were maintained on a chow diet and received 10% sucrose instead of water for one hour during the treatment. Finally, four male rats were trained in a sucrose detection task. Unless otherwise mentioned, all rats were kept in a temperature-controlled environment at 21 ± 1°C and on a 12:12 hour light-dark cycle (06:00–18:00).

#### Tesofensine administration on a diet-induced obesity model

This study investigated the effectiveness of tesofensine in treating obesity using a diet-induced obesity model [20, 21]. Three-week-old, weight-matched male rats (60–65 g) were housed in pairs and fed either a standard chow or high-fat diet until they reached 12 weeks of age. After 12 weeks, the rats were divided into four groups: Chow-Saline (n = 6), Chow-Tesofensine (n = 7), HFD-Saline (n = 6), and HFD-Tesofensine (n = 8). Rats were housed individually during the treatment and maintained access to their assigned diet. The two tesofensine groups received 2 mg/kg tesofensine, injected subcutaneously 60 minutes before the dark cycle onset for 15 consecutive days. The rat’s body weight and food intake were measured every 24 hours and expressed as daily body weight gain compared to the first day of drug administration. After the treatment, the rats were fasted for 12 hours and euthanized by an overdose of pentobarbital sodium anesthesia (100 mg/kg i.p.). Total fat mass was measured by weighing the gonadal, perirenal, and mesenteric adipose tissues [22–24].

#### Surgery for extracellular recording in rat’s LH

The electrophysiological data was collected and processed as detailed in extracellular recordings in mice. All rats underwent surgery under anesthesia, obtained by an intraperitoneal injection of xylazine (8 mg/kg) and ketamine (80 mg/kg). A local analgesic, lidocaine (4 mg/kg of 1% solution), was administered subcutaneously under the head skin. The rats were then placed in a stereotaxic apparatus for implantation of a homemade electrode array composed of 16 tungsten wires (35 μm in diameter, arranged in a 4x4 array with an area of 1 mm^2^) in the right LH (AP -2.1 mm, ML -1.5 mm from bregma, and DV -8.3 mm from the dura). The electrode array was attached to a dedicated tungsten filament inserted into the LH, and a stainless-steel screw was soldered to a silver wire for electric ground, which was screwed above the cerebellum and cemented into the skull.

For subcutaneous catheter implantation, the rats underwent two small incisions (∼1mm) in the superior left abdomen and dorsal neck areas. Sterilized silicone tubing (12 cm long, Silastic laboratory tubing, Dow Corning, Midland, MI, CAT. No. 508–004) was used as a catheter and tunneled subcutaneously from the back incision to the dorsal neck incision. The incisions were then sutured and closed using USP 3–0 (Atramat, Mexico). After surgery, the rats were treated with intraperitoneal enrofloxacin (10 mg/kg) and meloxicam (2 mg/kg) for three consecutive days. Experiments began seven days after surgery [25].

#### Histology in rats

Rats were anesthetized with an overdose of sodium pentobarbital (150 mg/kg), then perfused intracardially with PBS 1x and paraformaldehyde at 4%. The brain was removed and put in a 10% sucrose solution for 24 h, followed by sequential increases in sucrose concentration until reaching 30% in a 72-h period. The brain was then sliced coronally (50 μm thick) and stained with cresyl violet. For histological confirmation of electrode location in the brain, the electrodes were covered with DiI lipophilic carbocyanine dye (1%; Sigma-Aldrich) allowing the observation of the fluorescent track left by the electrodes.

#### Chronic treatment with tesofensine and 5-HTP/CB

The interaction between tesofensine and 5-HTP was characterized in lean male rats fed a standard chow diet. The rats were given daily injections of tesofensine plus a fixed dose of 5-HTP (31 mg/kg, i.p.) and CB (75 mg/kg, i.p.) for 15 days, following one of six protocols: vehicle (control, n = 6 for each group), 5-HTP/CB (31 and 75 mg/kg), tesofensine1 (1 mg/kg, s.c.), tesofensine2 (2 mg/kg, s.c.), tesofensine1 + 5-HTP/CB, and tesofensine2 + 5-HTP/CB. CB was administered 30 minutes before 5-HTP, and tesofensine was injected subcutaneously 30 minutes after 5-HTP. Body weight and food intake were measured every 24 hours and expressed as daily body weight gain or food intake relative to the first day of drug administration. The drugs were administered between 13:00 and 18:00 h.

#### Isobologram curve for sucrose intake assay

The pharmacological interaction between tesofensine and 5-HTP/CB was characterized by isobolographic analysis. Isobolographic analysis was implemented to determine if the interaction between two drugs given in combination is synergistic (supra-additive), additive, or antagonistic (infra-additive) [26, 27]. It is widely used for the evaluation of combinations of a variety of drugs, including analgesics [28–30], gastroprotective drugs [31], and anticonvulsants [28], among several other pharmacological agents.

Tesofensine and 5-HTP/CB anorexigenic effects were assayed as the volume of a sucrose solution consumed in one hour. The percentage of the anorexigenic effect on the experimental day was calculated by subtracting the observed to the basal intake and then dividing the result by observed intake, by the following formula [29–31]:

$$\%\;anorexigenic\;effect=\frac{Sucrose\;intake\;on\;basal\;period-Sucrose\;intake\;on\;experimental\;day}{Sucrose\;intake\;on\;experimental\;day}*100$$

Dose-response curves were constructed by plotting the % of anorexigenic effect against the tesofensine or 5-HCP/CB administered individually, and ED30 (+ SEM) values were estimated by linear regression. The interaction between tesofensine and 5-HTP/CB combinations in a 1:1 proportion was characterized as described by [29], while a 3:1 combination was analyzed as described by [30]. The used doses for the combinations were fractions of the ED30 values observed with the individual components. The actual doses of the combinations are shown in **Table 1**.

**Table 1:** The dose ratio of the co-administration of tesofensine + 5-HTP (mg/kg).

1:1	3:1
4.25	3.17
2.12	1.59
1.06	0.79
0.53	0.40

5-HTP/CB dose against tesofensine dose plots were constructed and an oblique line (isobole) was drawn by joining the ED30 values of the individual components. The theoretical ED30 value of the combination corresponding to a pure additive interaction is located on this line [29, 30]. The interaction index is estimated as the ratio of the experimental divided by the theoretical ED30, and the experimental ED30 is then compared to the theoretical value by the modified Student’s t-test [26]. An experimental ED30 statistically significantly lower than the theoretical ED30 is an indicator of a synergistic (supra-additive) interaction, whereas a significantly higher experimental value corresponds to an infra-additive interaction between the individual components. On the other hand, if no statistically significant difference between the experimental and theoretical ED30 values is detected, an additive interaction is concluded.

#### Homegustometer appararatus

A homegustometer is a homemade behavioral setup that adapts the animal homecage for psychophysical tasks. Rats can perform the task day and night for weeks. To create the homegustometer, the frontal wall of a standard homecage was opened to allow the insertion of three sippers. The central sipper was fixed in position, while the two lateral sippers were adjusted to 60 degrees relative to the central sipper. A metal floor was inserted into the homecage’s floor, serving as a ground for contact lickometers. All licks were recorded using a Med Associates interphase.

#### Sucrose detection task

To assess sucrose’s perception, rats were trained to visit a central port and give between 2 and 5 licks in an empty sipper to receive a 10 μL drop comprising either water or one of five sucrose solutions with varying concentrations (0.5, 1.3, 3.2, 7.9, or 20% w/v). Trials were balanced such that the probability of receiving water (0%) or sucrose (any concentration) was 0.5, and they were presented in pseudo-random order. Then the subjects were required to report whether the drop contained or did not contain sucrose, by approaching and then licking the left outcome port if the stimulus was water (0%), and the right port if it was sucrose. This rule was counterbalanced across subjects [32]. Successful detection led to reward, which consisted of the delivery of a drop of water per each of the subsequent three licks. Error trials were unrewarded. Trials ended 0.3 seconds after the last water drop for rewarded trials; and for unrewarded trials, the trials ended 0.3 seconds after the first dry lick. After receiving either the Stimulus or the Reward, the subjects could keep dry licking the ports with no penalties but wasting time to complete more trials and obtain more rewards. The number of dry licks after the Stimulus in the central port is an indirect measurement of the hedonic value of the tastant; indeed, in our task the post-stimulus licks increased with sucrose palatability [33]. For this reason, the task could measure oromotor palatability responses elicited by one single drop of sucrose.

The subjects were trained in the homegustometer which allowed a 23 h training in their homecages. Four rats were trained in the sucrose detection task for at least 10 days until they achieved 75% correct responses during two consecutive days. Following initial training (not shown), rats underwent 1–2 months break before being reintroduced to the homegustometer. Three baseline sessions were recorded before 5 days of treatment with s.c. tesofensine 2 mg/kg. This was followed by 3 days of saline injections in the post-tesofensine period. The equipment was cleaned and recalibrated daily at noon, and injections occurred around 18:00. Thus, rats had access to water *ad libitum* in their home cages for one hour while the homegustometer was being cleaned.

#### Open acrylic box and locomotor activity

For behavioral experiments, locomotor activity was measured in an acrylic box (41.5 cm in length, 30 cm in width, and 26 cm in height) coupled with a camera (in the bottom view position). From a bottom-view video recording, the animals’ position at x and y coordinates of rats’ noses, forelimbs, hind-limbs, and tail base was tracked using DeepLabCut software (DLC) [34]. A video was recorded at 60 frames per second (fps) with a resolution of 1280 x 720 pixels using a Kayeton camera (model KYT-U400-MCS2812R01). The forward locomotion was tracked using the rats’ center mass of the hind-limbs method and plotted as total distance traveled (cm) for 240 minutes.

### Data analysis

All data analysis was performed using MATLAB (The MathWorks Inc., Natick, MA), GraphPad Prism (La Jolla, CA, USA), DeepLabCut, and Python. For isobologram analysis we wrote a custom Matlab script that is available as supplementary material (IsobologramAnalysis.m). Unless otherwise indicated, we used the mean ± sem and the α level at 0.05.

#### DeepLabCut

DeepLabCut 2.2.3 was used to track all points of interest in a bottom-view videotape. A network was trained using 15 frames from 12 randomly selected videos for 1,030,000 iterations. The learning rate was decreased in a stepwise fashion, starting at 0.005 for 10,000 iterations, then 0.02 for 750,000 iterations, 0.002 for 800,000 iterations, and finally 0.001 for the remaining 103,000 iterations. Ten outliers from each training video were then corrected by relabeling points with a likelihood below 0.9. The network was then refined using the same number of iterations.

#### Measurements of head weaving stereotypy

The head weaving stereotypy was measured using the data obtained from DLC tracking of the angular variation of the Euclidean position of the nose regarding its base tail. Snippets were made from the angular variation data by averaging 3600 data points corresponding to one minute of the session time. Outliers were removed, and the first derivative of the snippet data was taken. Once this new vector was obtained, the standard deviation of the data was calculated, and it was found that the values greater than two negative standard deviations and less than two standard deviations and the centroid corresponding to the hind-limbs remain static (variation less than 1cm between minutes) corresponds to data describing the head weaving behavior. We consider stereotypy only for moments in which the rat remained immobile with four legs in contact with the floor [25]. These results were displayed as the percentage of time spent in each behavioral state.

#### Statistical analysis

For statistical analysis of differences between groups treated with tesofensine plus 5-HTP/CB, a repeated measure two-way ANOVA was conducted, followed by a Tukey’s *post hoc* test. The experimental Effective Dose (ED_30_) for tesofensine and ED_30_ for 5-HTP were found using linear regression of the log dose-response curves for the isobolographic analysis. The theoretical additive line was constructed by plotting the ED_30_ values of tesofensine and 5-HTP alone and by computing the theoretical ED_30_ of the combination. The dose-response curve was obtained after the co-administration of fixed doses of tesofensine and 5-HTP based on fractions (1/2, 1/4, and 1/8) relative to their respective ED_30_ values. A one-way ANOVA followed by a Dunnett’s test post-hoc was used in the GraphPad Prism 6 software. The dose ratios of tesofensine and 5-HTP were 1:1 and 3:1 and the experimental ED_30_ values of the combination were determined from the log dose-response curve for each dose ratio using linear regression. Statistical difference between each theoretical ED_30_ and its experimental ED_30_, along with confidence intervals for each dose ratio and its interaction indices, were calculated following the modified t-test proposed by [35], implemented in a homemade MATLAB script (available as supplementary material).

#### Electrophysiological data analysis

Statistical analysis and figures were performed using MATLAB (MathWorks). We used a chi-square test to assess differences in the proportion of neurons recruited. The Peri-stimulus Time Histograms (PSTHs) were then color-coded into z-scores.

t-distributed Stochastic Neighbor Embedding (t-SNE) is an automatic dimensionality reduction method that tries to group neurons with similar firing rates in a low-dimensional space to optimally preserve neighborhood identity [36]. In this manuscript, t-SNE was used to reduce the dimensionality of the matrix with neuronal activity. All data points were grouped using a hierarchical clustering analysis running the Matlab function *linkage (Ward)*. The concatenated matrix of all neurons was used to classify them into one of four mathematical “clusters,” now called “ensembles.” An “Elbow curve” method was used to find the optimal number of ensembles. The data were separated into different numbers of putative ensembles, ranging from 2 to 15. The distance of each neuron to the centroid of their respective cluster was then calculated. As the number of ensembles increased, the distances to the centroid of each ensemble were reduced. A curve was then created by plotting the total distance within each ensemble against the number of ensembles tested. The elbow point of this curve was where the slope decreased below 0.5. The number of ensembles at the elbow point indicated a recommended number, reflecting a balance between a low intra-ensemble distance and a high number of ensembles. In our data, the elbow was found in 4 ensembles. We have previously used a similar approach but are using PCA rather than t-SNE [37].

We also used t-SNE to analyze the profile of motor effects induced by appetite suppressants, in this case, clustering rats exhibiting similar motor side effects.

## Results

### Tesofensine demonstrated greater weight loss efficacy in obese rats

**Fig 1:**
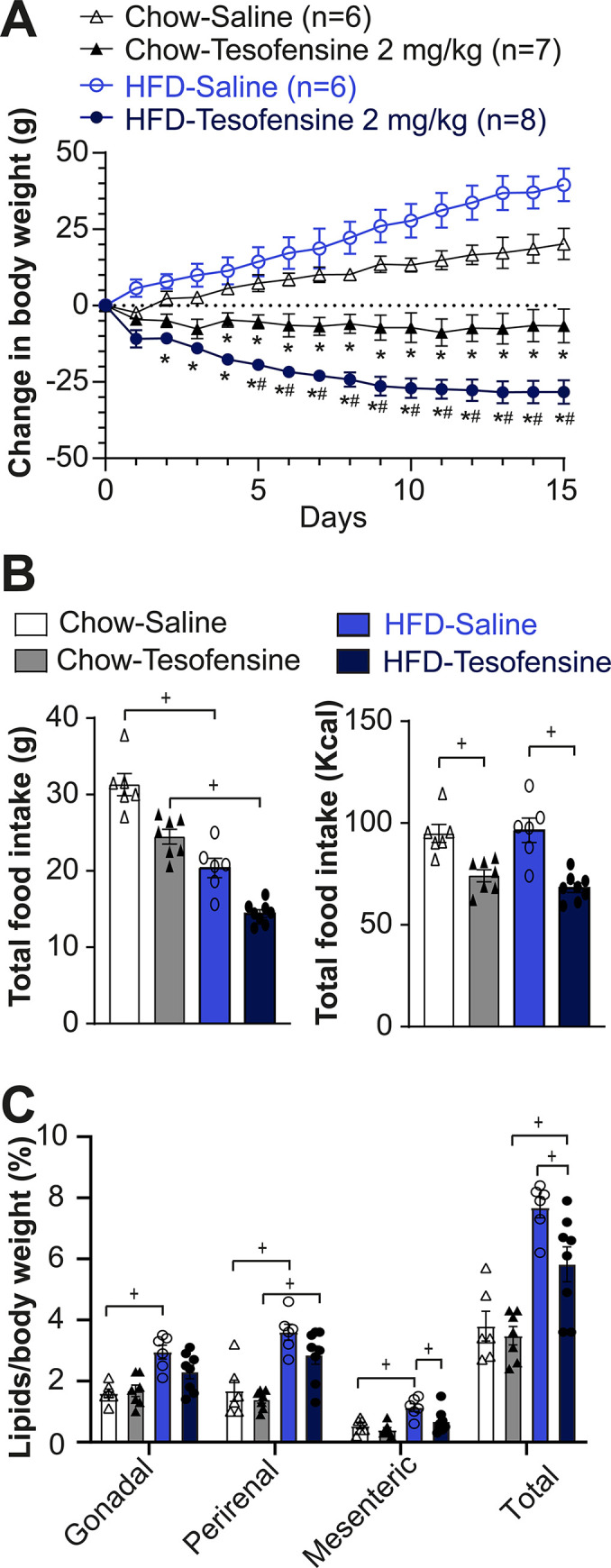
Tesofensine induces greater weight loss in HFD-fed rats compared to chow-fed rats. A. Change in body weight across treatment days with the treatment groups receiving subcutaneous injections of tesofensine (2 mg/kg) and the control groups receiving saline. B. Total grams of food consumed per group (left panel) and total caloric intake (right panel). Both groups treated with tesofensine consumed fewer calories than saline control groups. C. Visceral fat content (%) relative to body weight in gonadal, perirenal, and mesenteric deposits. Data are presented as mean ± SEM. n = number of rats. Filled and open data points represent male rats (B-C). Repeated Measures ANOVA (A), One-way ANOVA (B-C). * p <0.05 compared with Chow-Saline, # p < 0.5 compared with Chow-Tesofensine and + p < 0.05 significantly different between groups.

First, we asked whether tesofensine has the same efficacy in obese and lean rats. To do this, we characterized the anorexigenic and body weight loss effects induced by tesofensine in both types of rats. Rats had ad libitum access to a standard chow or a high-fat diet for 12 weeks during childhood and adolescence. Obese rats fed with a high-fat diet weighed more initially (588.7 ± 14.1 g) than rats on a standard chow diet (497.8 ± 15.1 g, *p* < 0.05) (**S1 Fig**). Every 24 hours, we weighed the daily food intake and body weight and subsequently injected tesofensine or saline. A constant weight gain was observed in Chow-Saline and HFD-Saline groups (**Fig 1A**). In contrast, after three days of treatment, Chow-Tesofensine and HFD-Tesofensine groups decreased body weight compared to control rats receiving saline. Afterward, the HFD-Tesofensine group lost weight faster than Chow-Tesofensine-treated rats. Lean rats treated with tesofensine reached a plateau after the third day until the end of treatment and demonstrated less body weight loss than obese animals treated with tesofensine. This study found that tesofensine was more effective in inducing weight loss in obese rats than in lean rats.

The weighed food intake is shown in **Fig 1B**, left panel. Since diets have different amounts of energy, we normalized them to kilocalories (**Fig 1B,** right panel). It can now be seen that tesofensine reduced the total energy intake in both Chow-Tesofensine and HFD-Tesofensine treated groups compared with control rats receiving saline (**Fig 1B,** right panel). We observed no significant difference in kilocalories consumed between Chow-Tesofensine vs. HFD-Tesofensine groups (*p* = 0.6). The results suggest that the increased effectiveness of tesofensine in promoting weight loss in obese rats could be because of an increase in energy expenditure rather than its anorexigenic effects.

Next, we quantified the effect of tesofensine on the visceral fat proportion of body weight in lean and obese rats. We found a significant difference in total visceral fat (composed of gonadal, perirenal, and mesenteric fat) between the HFD-Saline and HFD-Tesofensine groups (**Fig 1C**). However, the total fat in the Chow-Tesofensine group did not differ significantly from that of the Chow-Saline group. These results indicate that tesofensine reduced total visceral fat, mainly mesenteric fat deposits, in obese rats.

### Tesofensine-induced modulation of lateral hypothalamic neurons is more pronounced in obese than in lean rats

The LH plays a vital role in seeking food and regulating feeding behavior [5, 11, 13]. It is believed to be a primary target for various appetite suppressants, and recently, it was found that tesofensine could be a potential treatment for hypothalamic obesity, a rare feeding disorder [1, 38, 39]. Hence, LH might be a potential target of this drug.

**Fig 2:**
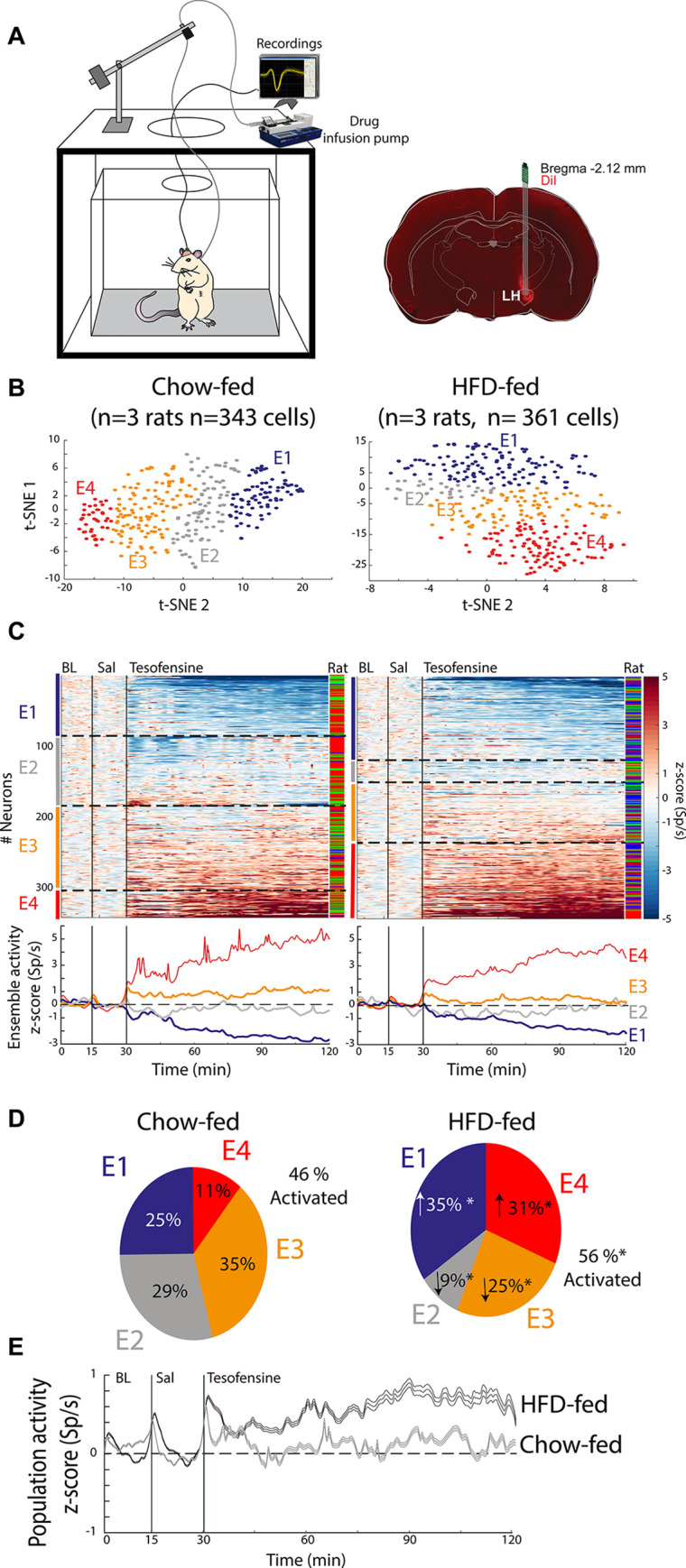
Tesofensine differentially modulates lateral hypothalamic neurons in lean and obese rats. A. Left: A schematic representation for extracellular recording in a freely moving rat and pump for automatic drug delivery. No food was available during recordings. Right: histology, identifying recording sites in LH using a coronal section. Dil, a fluorescent and lipophilic red dye, is used to mark the tip of the electrode track. B. t-SNE and hierarchical cluster analysis of firing rates were used to group neurons with similar activity patterns into neuronal ensembles. This analysis uncovered four ensembles responding to tesofensine. Neurons were assigned into four ensembles (E1-4) for Chow-fed rats (left) and four ensembles for HFD-fed rats (right). Each dot represents a single neuron and the color of the ensemble to which it belongs in the t-SNE map. C. All neurons recorded were categorized into four ensembles (E1-E4) for Chow-fed rats (left) and HFD-fed rats (right). The normalized color-coded activity of each neuron over time is presented for chow-fed and HFD-fed rats, with red and blue indicating higher and lower z-score activity, respectively. Black vertical lines show the baseline (BL from 0–15 minutes), saline (Sal 15–30 minutes), tesofensine administered at 30 minutes and recordings that lasted up to 120 minutes. In three HFD rats, we recorded n = 127, 95, and 139 neurons, whereas in the other three Chow-fed rats, n = 220, 106, and 17 neurons. The color bar on the right indicates the rat in which the neuron was recorded. Peri-Stimulus Time Histograms (PSTHs) below demonstrate the average neuronal ensemble activity, with dashed lines dividing each cluster (ensemble). D. Pie charts depict the percentage of neurons in each ensemble for Chow-fed and HFD-fed rats. Chi-square analysis showed a significant difference compared to the same ensemble in Chow-fed rats (* p<0.05). E. The z-score normalized population activity of all lateral hypothalamic neurons recorded in HFD-fed (n = 361) and chow-fed rats (n = 343) is presented. The shadow represents the SEM.

To examine this possibility, we evaluated whether tesofensine exerts a differential effect on LH cell activity of lean versus obese subjects. Thus, we conducted multichannel recordings in the LH of six rats, three fed with a standard diet and three high-fat diet. During the recordings in the LH, we administered saline and tesofensine subcutaneously via a catheter. A total of 343 neurons in the Chow-fed rats and 361 in HFD-fed rats were recorded (**Fig 2A**). We then used a t-distributed stochastic neighbor embedding (t-SNE) to classify the firing rates of recorded neurons into ensembles. The results are shown in **Fig 2B**, with the left panel depicting neurons recorded in Chow-fed rats and the right panel for neurons recorded in HFD-fed rats. **Fig 2C** shows the neuronal responses over two hours of recordings. The normalized firing rates were then color-coded, with blue indicating decreased and red increased activity. t-SNE and hierarchical clustering analysis unveiled four ensembles: The first ensemble (E1) includes a cluster of neurons that exhibited a robust inhibition lasting 1.5 hours after the tesofensine administration. The second neuronal ensemble (E2) showed modest inhibition in activity. In contrast, the third and fourth ensembles showed modest (E3) and substantial (E4) neuronal responses (activation), respectively. The proportion of neurons in each ensemble differed significantly between obese and lean rats. Specifically, the proportion of neurons inhibited by tesofensine (E1) increased from 25% (86/343) in Chow-fed rats to 35% (125/361) in HFD-fed rats (Chi-square = 7.19, *p* = 0.0073). Similarly, tesofensine recruited a greater proportion of activated neurons (E4) in obese rats (31%, 113/361) than in lean rats (11%, 38/343) (Chi-square = 41.50, *p*<1.1771e-10) (**Fig 2D**). These findings indicate that administering tesofensine to rats exposed to a high-fat diet during childhood and adolescence resulted in a different modulation pattern in the LH compared to age-matched lean rats. In addition, the recorded population activity of neurons demonstrated a bias towards activation in obese rats but not in lean rats (**Fig 2E**). For example, the sum of ensembles 3 and 4, which exhibited increased firing rates after the tesofensine administration, was 56.2% (E3 = 90 + E4 = 113; 203/361) HFD-fed rats, compared to 46% (E3 = 120 + E4 = 38; 158/343) in Chow-fed rats (Chi-square = 6.87, *p* = 0.0087). Our analysis found that the differences between the effects of tesofensine on the LH ensemble activity of chow-fed and HFD-fed rats were statistically significant. These findings indicate that tesofensine effect over LH modulation is different in obese rats chronically exposed to a high-fat diet during childhood and adolescence, compared to lean subjects: tesofensine promotes the recruitment of cells belonging to strong-inhibitory (E1) and -excitatory (E4) responsive ensembles, and an increased sustained overall LH population activity.

### Tesofensine silenced LH GABAergic neurons in transgenic mice

**Fig 3:**
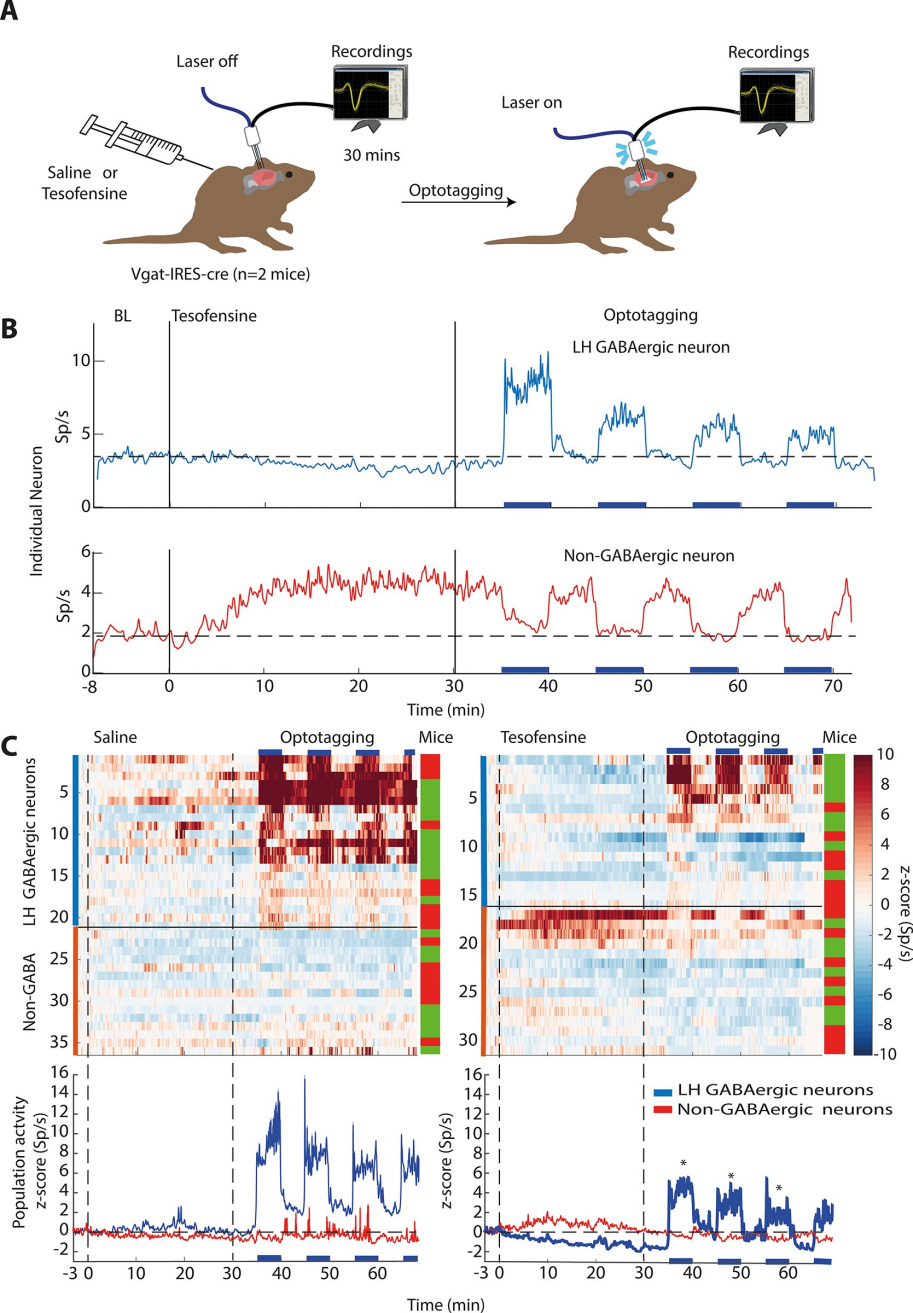
Tesofensine silences mice LH GABAergic neurons and attenuates their optogenetic activation. A. Extracellular neuronal activity was recorded in two mice over a baseline period of 5 minutes before they received subcutaneous injections of saline or tesofensine (2 mg/kg). After 30 minutes, a laser was activated on an open loop protocol. It started with 5-minute blocks of inactive periods (no laser) and active (50 Hz; 2s on + 4s off). Employing a within-subjects design, the same two mice received injections in this order: tesofensine (day 1), saline (day 2), tesofensine (day 3), and saline (day 4). We combined the activity of neurons recorded on both days of tesofensine for further analysis, and the same was done for saline days. This design allowed each mouse to serve as its own control. While we cannot guarantee recordings were done in the same neurons across days due to technical limitations, however, we used fixed arrays to maintain the electrode position throughout the four recording days. B. Upper depicts responses of single GABAergic neurons inhibited after tesofensine administration. Bottom a non-GABAergic neuron. C. A heat map displaying neuronal activity during saline (left) and tesofensine (right) conditions. Blue horizontal lines indicate stimulation blocks. Below, the corresponding peri-stimulus time histogram (PSTH) shows the average activity of neurons during the stimulation blocks, with the dashed line representing a zero z-score. * GABAergic neuron activity was significantly reduced by tesofensine compared to saline treatment [RM ANOVA a significant treatment effect; F(1,35) = 6.012 p = 0.0193, and significant effect of block (3 stimulation blocks), F(1,2) = 10.6 p = <0.001, and no significant interaction F(2,70) = 0.02, p = 0.977]. Thus, tesofensine reduced neuronal activity even during optogenetic stimulation, highlighting its ability to silence GABAergic neurons in the LH. In contrast, non-GABAergic neuron activity showed no significant difference between saline and tesofensine groups (p > 0.05), as assessed by an RM ANOVA [treatment main effect, F(1,28) = 0.25, p = 0.8754].

Following the observation of distinct effects of tesofensine on LH activity in obese and lean rats, we investigated the specific cell type in this region that was primarily affected by the drug in mice. We hypothesize that tesofensine could affect GABAergic neurons due to its role in seeking and consummatory behaviors [11, 13]. To optogenetically identify LH-GABAergic neurons, we perform optrode recordings in lean Vgat-IRES-Cre mice, as depicted in **Fig 3A**. We recorded LH multichannel activity during a baseline period of at least 5 minutes before injecting saline or tesofensine 2 mg/kg subcutaneously on alternating days. After a minimum of 30 minutes, we conducted an optotagging assay comprising 5-minute blocks of active (50 Hz and laser turned 2s on, 4s off) and inactive periods. **Fig 3B** shows the firing rates of two distinct LH individual neurons. The first neuron exhibited a gradual decrease in firing rate following tesofensine administration. During the optotagging epoch, we identified it as GABAergic because it showed increased activity during the 5-minute block of photostimulation. Conversely, the second example is a non-GABAergic neuron because it was inhibited during photostimulation. Additionally, it exhibited a significant increase in firing rates following tesofensine administration. **Fig 3C** shows the color-coded activity of all neurons opto-identified as GABAergic and non-GABAergic and their population activity. During saline injection days (left panel), neither GABAergic nor non-GABAergic neurons were modulated after saline injection. During optotagging (see 30–66 minutes), only GABAergic neurons (blue trace) responded during laser stimulation.

During sessions with tesofensine administration (right panel), we observed that the population activity of GABAergic neurons was gradually inhibited after tesofensine administration (blue trace, see 0–30 minutes). Moreover, the population responses elicited by the optogenetic stimulation (optotagging see 30–66 minutes) were significantly weaker under tesofensine treatment than saline injection (compare the blue PSTH lines in the right vs. left panels, respectively, see * in **Fig 3C**). In contrast, the non-GABAergic neurons (red trace) showed a slight increase in activity after tesofensine administration (see 0–30 mins). They were either inhibited or not modulated during the optotagging assay (see 30–66 mins). Thus, tesofensine silences a subset of LH GABAergic neurons and attenuates their open loop optogenetic activation.

### Tesofensine reduced feeding behavior induced by optogenetic activation of LH GABAergic neurons in lean Vgat-ChR2 mice

**Fig 4:**
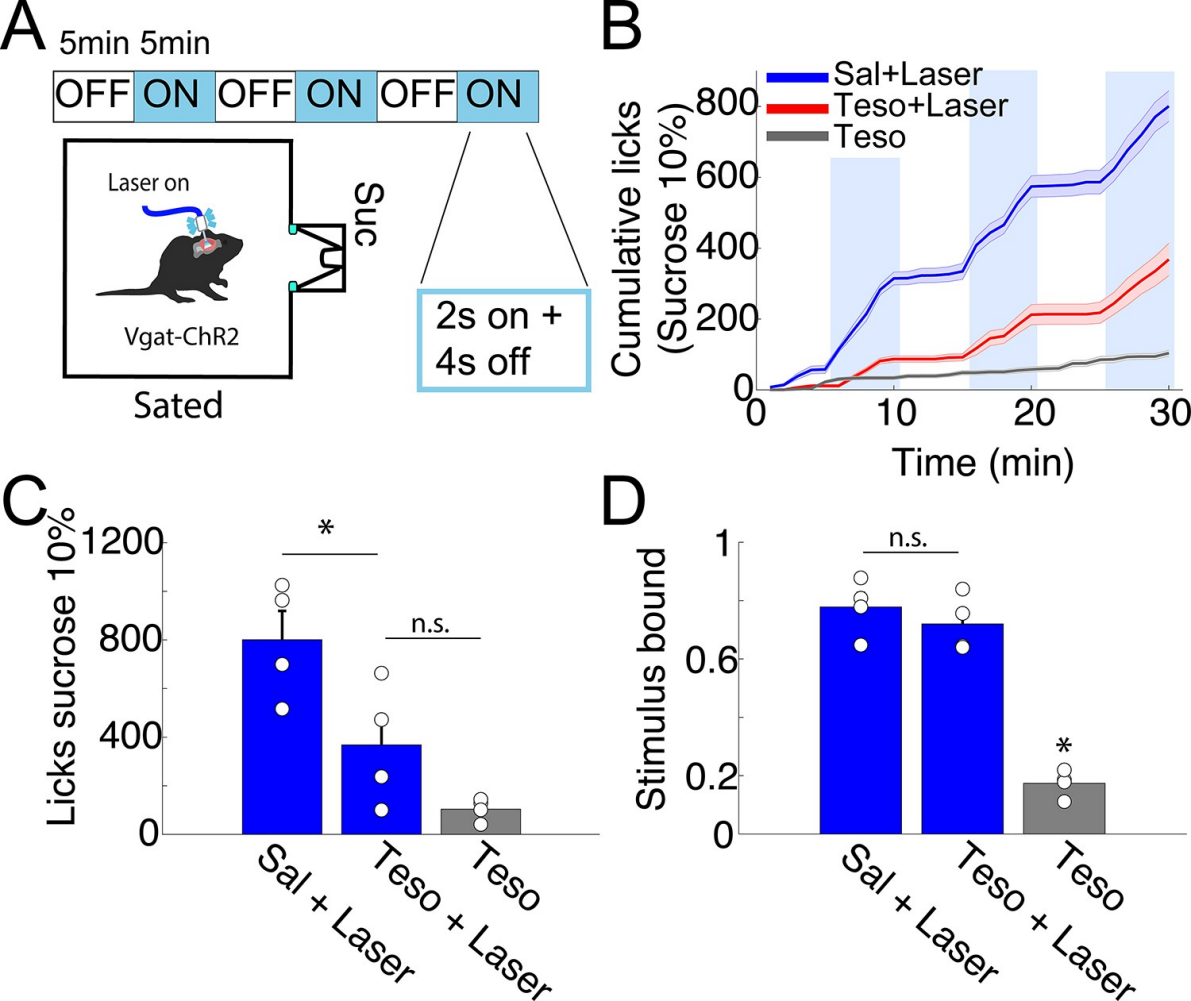
Tesofensine reduces the feeding behavior induced by the optogenetic activation of LH GABAergic neurons in lean Vgat-ChR2 mice. A. Schematic of the open-loop task, where VGAT-ChR2 mice (n = 4) with a fiber implanted in their LH, were stimulated at 50Hz for 2 seconds with 4 seconds off in blocks of 5 minutes (blue). Mice could lick a sucrose solution (10%) from a sipper. B. Cumulative licks during the open-loop stimulation with tesofensine (Teso) 2mg/kg or saline solution (Sal) administered by subcutaneous injection 30 minutes before the task. The tesofensine group (gray) was placed in the task, but the laser was not stimulated. Note that the teso+laser group exhibited an attenuated feeding response elicited by stimulation of LH GABAergic neurons. C. Total licks during a session, with blue bars representing the blocks that were laser stimulated in the task. D. Stimulus-bound feeding is the fraction of licks given during the stimulation window (spanning from the first laser pulse to 2.5 seconds after), with each circle representing a different mouse. Data are presented as mean ± SEM, and the results were statistically significant (*p-value < 0.05 based on paired t-test value).

Based on the findings that tesofensine attenuates LH-GABAergic activity and optogenetic stimulation of these cells promotes sucrose consumption in sated mice [8, 11], we investigated whether tesofensine would alter sucrose consumption induced by LH-GABAergic population activation in lean mice. To achieve this goal, we used sated transgenic Vgat-ChR2 mice (n = 4) that constitutively expressed the opsin channelrhodopsin (ChR2) in GABAergic neurons to optostimulate in 5 minutes on and off blocks [11]. On different days, we injected saline, tesofensine 2 mg/kg + laser, or tesofensine 2 mg/kg alone without optostimulation (**Fig 4A**). The cumulative number of licks given in 30-minute session can be seen in **Fig 4B.** Under saline injection, the Vgat-ChR2 mice increased sucrose intake selectively during optostimulation (see blue line). In contrast, under tesofensine treatment, the licks evoked by optostimulation of LH GABAergic neurons diminished (**Fig 4C**, see saline injection + laser vs. tesofensine+laser). These results indicate tesofensine attenuated open loop induction of sucrose feeding.

In addition, it is well known that LH GABAergic stimulation typically leads to stimulus-bound feeding. Most feeding occurs within 2.5 seconds of optogenetic stimulation [11] (**Fig 4D**; Sal + laser). In an open loop protocol (i.e., independently of behavior), we found that tesofensine treatment reduced the number of licks but did not affect stimulus-bound feeding (**Fig 4D**, Teso + Laser), showing that the drug per se did not impair oromotor reflexes elicited by optogenetic stimulation. These results demonstrate that the tesofensine-induced reduction in sucrose consumption, measured by the number of licks, is due to decreased feeding consummatory behavior rather than simply impairing oromotor reflexes elicited by optogenetic stimulation.

### LH GABAergic neurons in obese mice might be more potent in driving feeding behavior than those in lean mice, under tesofensine treatment

**Fig 5:**
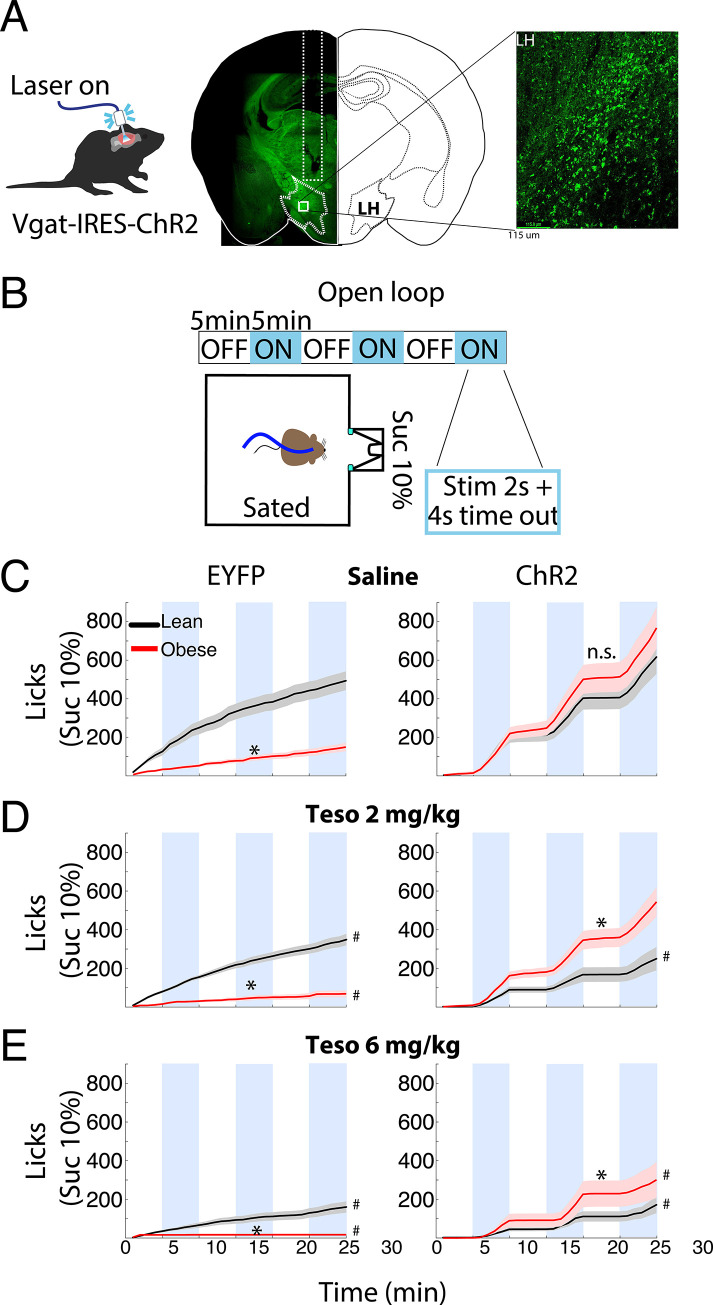
Tesofensine does not attenuate the optogenetic feeding elicited by LH GABAergic neurons in obese Vgat-IRES-cre mice. A. Representative image of Vgat-IRES-Cre mouse expressing ChR2 neurons (green) in the LH. B. Behavioral protocol: Vgat-mice were fed an HFD or standard Chow diet for 12 weeks and were optostimulated in an open loop schedule. The mice had access to a sucrose solution (10%) in a sipper during the task. Mice had food and water ad libitum before the task. C-E. These six panels depict the cumulative licks (intake) during the open-loop task for lean versus obese mice administered with saline (C), tesofensine (Teso) 2 mg/kg (D), or 6 mg/kg (E) 30 minutes before the task. The left panels are control mice expressing EYFP, and the right panels express ChR2. Data are presented as mean ± SEM. The results show that compared to saline, tesofensine 2 mg/Kg reduced sucrose intake in lean mice expressing ChR2 (D right panel, see black line). In contrast, activating GABA neurons in obese mice significantly induced more sucrose intake than in lean mice.* p <0.05, Two-sample Kolmogorov-Smirnov test Lean vs. Obese. # p < 0.05 compared to its saline control group.

After demonstrating the anorexigenic effects of tesofensine in lean Vgat-ChR2 mice, we aimed to replicate our findings in obese Vgat-IRES-cre mice. We expressed ChR2 in the LH through viral infection and exposed the mice to a high-fat diet or standard chow for 12 weeks (**Fig 5A**). We optogenetically stimulated LH GABAergic neurons in an open loop optogenetic stimulation paradigm and measured sucrose intake by drinking through a sipper filled with sucrose (**Fig 5B**). As a control, we injected an EYFP-expressing AAV vector.

In **Fig 5C**, the left panel showed the cumulative licks in EYFP-expressing control mice and the right panel for ChR2-expressing mice. In the left panel, we can see the normal sucrose intake (in cumulative licks) of Lean-EYFP mice under saline injection. **Fig 5C** (left panel) shows the cumulative licks of EYFP-expressing control mice, while the right panel focuses on ChR2-expressing mice. Under saline injection, the left panel reveals the normal sucrose intake (cumulative licks) of sated Lean-EYFP mice. Paradoxically, we found that the control obese mice expressing EYFP (Obese-EYFP) consumed less sucrose overall compared to the Lean-EYFP mice (Two-sample Kolmogorov-Smirnov test, *p*<0.05), indicating that the HFD access may have indirectly led to sucrose devaluation. We do not know the reason for this phenomenon, but similar devaluation phenomena have been previously reported [40]. In contrast, optogenetic activation of LH GABA neurons in both Lean-ChR2 and Obese-ChR2 mice elicited a strikingly similar boost in sucrose intake confined to the stimulation blocks (**Fig 5C**, right panel). LH GABAergic neurons failed to differentially impact sucrose intake in Lean vs. Obese ChR2 mice under saline (*p* > 0.05, n.s.). Moreover, tesofensine’s ability to suppress sucrose intake in Lean-EYFP mice progressively amplified with dosage. **Fig 5C** (see #) reveals a significant reduction in cumulative sucrose licks at 2 mg/kg compared to saline, followed by an even steeper decline at 6 mg/kg (**Fig 5D**, see #). These findings conclusively demonstrate the dose-dependent anorexigenic properties of tesofensine in this model.

As expected, in Lean ChR2 mice, optogenetic activation of LH GABAergic neurons triggered a binge in sucrose intake (**Fig 5C**, see blue line). Remarkably, at both doses, tesofensine effectively suppressed this feeding response, significantly reducing cumulative licks compared to saline (**Fig 5C** and **5D**, see #). These findings showcase the anorexigenic potential of tesofensine in modulating LH GABA-driven feeding.

Surprisingly, in Obese ChR2 mice (**Fig 5D**, red trace), the 2 mg/kg dose of tesofensine failed to dampen the surge in sucrose intake triggered by optogenetic stimulation compared to saline (p<0.05, n.s.), whereas the higher 6 mg/kg dose did effectively suppress feeding relative to saline injection (see #). The direct comparison between ChR2 mice revealed an important difference between lean and obese mice (Lean-ChR2 vs Obese-ChR2; p’s < 0.05, see *) that is Obese-ChR2 mice consumed more sucrose than Lean-ChR2 mice under tesofensine treatment. This unexpected divergence suggests that GABAergic neurons in obese mice might be more potent in driving feeding behavior than those in lean mice, under tesofensine treatment, offering a potential explanation for the observed resistance to tesofensine at the lower (and at the higher **Fig 5E**) dose.

### The anorexigenic effects of tesofensine are amplified by the chemogenetic inhibition of LH GABAergic neurons

**Fig 6:**
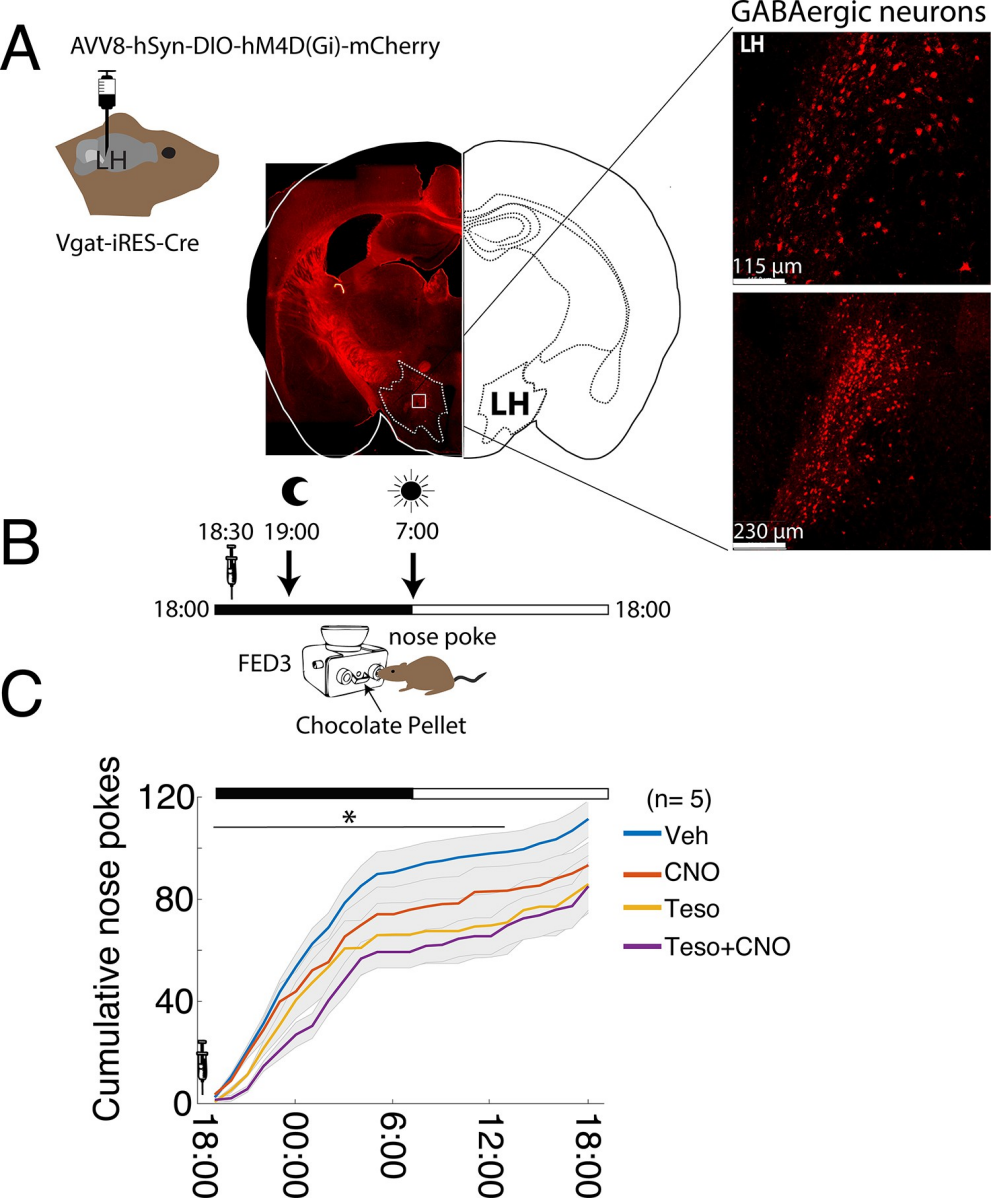
Chemogenetic silencing of LH GABAergic neurons potentiates tesofensine’s anorexigenic effects. A. Representative image of LH in mouse expressing hM4D(Gi), which inhibits GABAergic neurons chemogenetically. B. Schematic of a task where mice were placed in their home cages with an automated feeder (FED3) that delivered chocolate pellets for each nose poke. Recording started at 18:00 and lasted for 24 hours. At 18:30 h, the same five mice were injected with vehicle (Veh), Clozapine-N-Oxide (CNO), a ligand for hM4D(Gi), tesofensine (2 mg/kg), and CNO + tesofensine. The dark cycle lasted from 19:00 to 7:00 h. The moon symbol indicates when the ambient lights were turned off and the sun when the lights were turned on. C. Average cumulative nose pokes during the 24-hour recordings for each group. The syringe indicates the time of drug administration at 18:30. The teso+CNO group performed fewer cumulative nose pokes and thus obtained fewer pellets than the other groups. * Significantly different two-sample Kolmogorov-Smirnov test (for the first 19 hours after drug administration, see horizontal line); Veh vs. CNO p = 0.006, CNO vs. Teso; p = 0.01, and Teso vs. Teso+CNO p = 0.04.

We then tested whether inhibiting LH GABA neurons could enhance the anorectic effects of tesofensine (2 mg/kg). To reach this end, we employed a FED3 system to enable mice to obtain chocolate pellets via nose-pocking in their homecages while chemogenetic inhibition was performed on the GABA neurons. We used the Vgat-IRES-cre mice to virally express hM4D(Gi) in the LH, an inhibitory DREADD (**Fig 6A and 6B**). Using this DREADD, the evoked action potentials are inhibited on clozapine-induced activation of hM4D(Gi) [41]. Clozapine-N-Oxide (CNO) infusion occurred at 18:30, and nose pokes and pellets delivered were continuously recorded for up to 24 hours. Our results revealed that the combination of Teso+CNO resulted in greater cumulative feeding suppression than any individual treatment during the first 19 hours post-treatment (**Fig 6C**, Two-sample Kolmogorov-Smirnov test; Veh vs. CNO *p* = 0.006, CNO vs. Teso; *p* = 0.01, and Teso vs. Teso+CNO *p* = 0.04). Thus, silencing LH GABAergic neurons enhanced the anorectic effects of tesofensine.

### Comparison with other appetite suppressants in lean rats

Having shown the neuronal correlates of tesofensine in the LH in rats and mice, we compared tesofensine appetite suppressant effects with other appetite suppressants, particularly phentermine and 5-HTP.

#### Tesofensine induces body weight loss in rats without producing head weaving stereotypy at therapeutic doses

**Fig 7:**
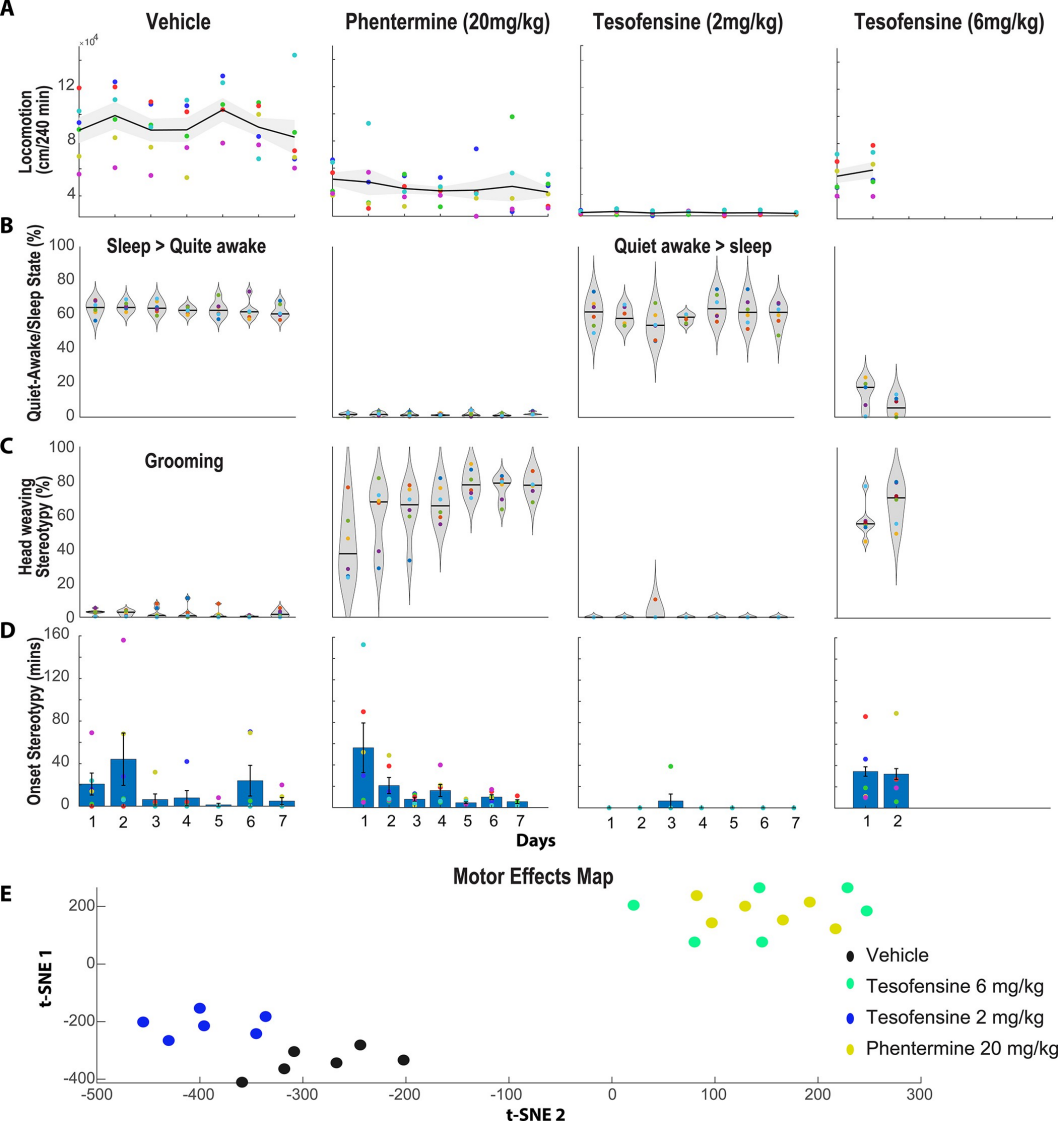
Effects of tesofensine and phentermine on locomotion, quiet-awake/sleep, and head weaving stereotypy in rats. A. Total distance traveled (cm/240 min) during forward locomotion over seven days of treatment with saline (1 ml/kg, n = 6), phentermine (20 mg/kg, n = 6), tesofensine (2 mg/kg, n = 6), or tesofensine (6 mg/kg, n = 6). Each point depicts one rat, with the black line indicating the mean and the gray shaded area indicating the standard error of the mean distance traveled by all rats in the group. All treatments reduced locomotion B. The proportion of time that rats spend in a quiet-awake/sleep state is defined as when the rat is not moving but either awake or sleeping. Only the Vehicle and Tesofensine 2 mg/kg groups spent more than 60% of their time in these behavioral states. C. The distribution of the percentage of time that rats exhibited a head weaving stereotypic behavior over seven days following treatment injection. Each point in the plot represents one rat, and the width of the violin plot indicates the probability density distribution. The black line shows the median value for each day. For phentermine, the results showed that the percentage of time the rat exhibited head weaving stereotypy gradually increased across the seven days. Additionally, head weaving stereotypy was aggravated across days in all rats treated with phentermine. In Vehicle control rats, grooming behavior was mistakenly classified as head weaving stereotypy. This is because control rats do not express this behavior. Surprisingly, rats treated with tesofensine 2 mg/kg exhibited little stereotypy, thus apparently neither grooming. D. The onset of the first event of stereotypy was measured across days. Note that for phentermine, the onset of stereotypy decreases across days. E. For the first two days, the variables locomotion, quiet awake/sleep, onset, and stereotypy were analyzed using a clustering algorithm (t-SNE). This analysis uncovered two main clusters: the first corresponds to rats treated with vehicle and tesofensine 2 mg/kg. Note that rats treated with tesofensine 2 mg/kg were in a slightly different position than control rats. The second cluster mixed rats treated with phentermine and tesofensine 6 mg/kg. Hence, t-SNE seems to separate rats according to the overall motor profile effects induced by each drug. At therapeutic doses, tesofensine induced body weight loss without producing head weaving stereotypy.

It has been proposed that tesofensine has an important dopaminergic component [3, 4, 42]. Hence, the motor effects of tesofensine were compared against phentermine, a hallmark dopamine-acting appetite suppressant. Involuntary movements with no apparent function are named stereotypies [43, 44]. Our research group recently reported that head weaving stereotypy is a common side effect of most appetite suppressants, particularly those acting to enhance DA efflux, such as phentermine [15, 25]. Unnecessary head oscillations indicated head weavings. Therefore, we characterized the tesofensine-induced stereotypy effects compared with phentermine, an amphetamine congener that served as a positive control. To quantify stereotypic behavior, we used DeepLabCut, a markerless pose estimation tool based on transfer learning with deep neural networks [34]. We trained the network to detect a rat’s nose, forelimbs, and tail base from a bottom-view videotaped session (see **S1 Video**). The video recording lasted for four hours after the drug administration. We observed that the control rats treated with saline exhibited a physiological level of forward locomotion (**Fig 7A**). Likewise, they spent about 65% of the session in a quiet-awake state (refer to **S1 Video**), most often in a "sleeping" position (**S2 Video**), which we pooled together for analysis (**Fig 7B**). Our algorithm incorrectly identified "head weaving stereotypy" in control rats, as these animals did not exhibit this behavior. This is because our algorithm identified a part of the grooming sequence and misclassified it as stereotypy (refer to **S3 Video** and [45]), likely because grooming and head weaving share certain similarities (**Fig 7C**). Nonetheless, this “grooming” behavior occurred randomly with low probability (**Fig 7C**; Vehicle, i.p.) and with variable onset times (**Fig 7D**).

Phentermine via i.p. resulted in a slightly increased locomotion and decreased time spent in a quiet-awake/sleep state (**Fig 7A and 7B**; Phentermine). Interestingly, DeepLabCut analysis unveiled for the first time that phentermine-treated rats exhibited less forward locomotion than control rats (despite it being a stimulant drug; **Fig 7A**). Notably, phentermine induced strong head weaving stereotypy, which increased gradually over seven days and occupied 80% of the time of the 4-hour session (**Fig 7C**). Head weaving stereotypic behavior involved rats standing still on four legs and moving their head erratically (**S4 Video**), accompanied by frequent uncontrolled tongue movements (although we did not formally quantify tongue movements, we report them as a subjective human visual observation). The onset of stereotypy decreased from 56.1 ± 23.2 minutes on the first day to 5.5 ± 1.8 minutes on the seven days of treatment (**Fig 7D**).

In contrast, at a low dose of tesofensine (2 mg/kg) induced little or no forward locomotion (**Fig 7A**). Rats spent more time in a quiet-awake state **(S5 Video)** than in a sleep position (**Fig 7B, S6 Video**), and head weaving stereotypy was detected in only one rat and for a short period (**Fig 7C;** day 3, **S7 Video**). As noted, our algorithm in control rats erroneously misclassified grooming behavior as stereotypy in control rats. However, no head weaving stereotypy was detected under tesofensine 2 mg/kg, suggesting, at least indirectly, a decrease in the probability of grooming behavior. Nevertheless, in rare instances, we observed that rats in a quiet-awake state would also execute jaw and tongue movements, albeit at a lower intensity (see **S8 Video**). Further studies are needed to evaluate these effects more carefully.

Finally, a high dose of tesofensine (6 mg/kg) was administered for two days only to avoid lethality, which led to increased locomotion and reduced time spent in a quiet awake/sleeping state (**Fig 7A and 7B**). At this high dose, rats exhibited clear and robust stereotypy behavior with rapid onset (**Fig 7C and 7D**), primarily comprising uncontrolled tongue movements and less intense head waving (**S9 Video**). From a visual inspection, we note that the stereotypy induced by tesofensine differs slightly from that induced by phentermine. However, both drugs share the common feature of inducing uncontrolled tongue movements, which earlier studies had failed to report. In summary, tesofensine at a low dose induced almost no head weaving stereotypy, but a robust stereotypy was observed at a high dose.

The motor map effects were represented using t-SNE (**Fig 7E**). This algorithm clusters rats’ behavior based on their overall profile of changes in motor variables, including locomotion, quiet awake/sleep time, onset, and stereotypy. Each dot on the map is a rat, and rats with similar profiles are grouped together. We observed that rats treated with tesofensine 2 mg/kg exhibited different behavior compared to the control group. In contrast, rats treated with tesofensine 6 mg/kg and phentermine, which both exhibited more stereotypy, were grouped in a small area but far away from the rats in the control and tesofensine 2 mg/kg groups (**Fig 7E**). Further studies are needed to investigate the effects of tesofensine on reducing the likelihood of grooming behavior and other tongue kinematics parameters.

### Pharmacological interaction with a serotonin appetite suppressant

#### Tesofensine prolongs the body weight loss effect of 5-HTP/CB, a serotonin precursor

**Fig 8:**
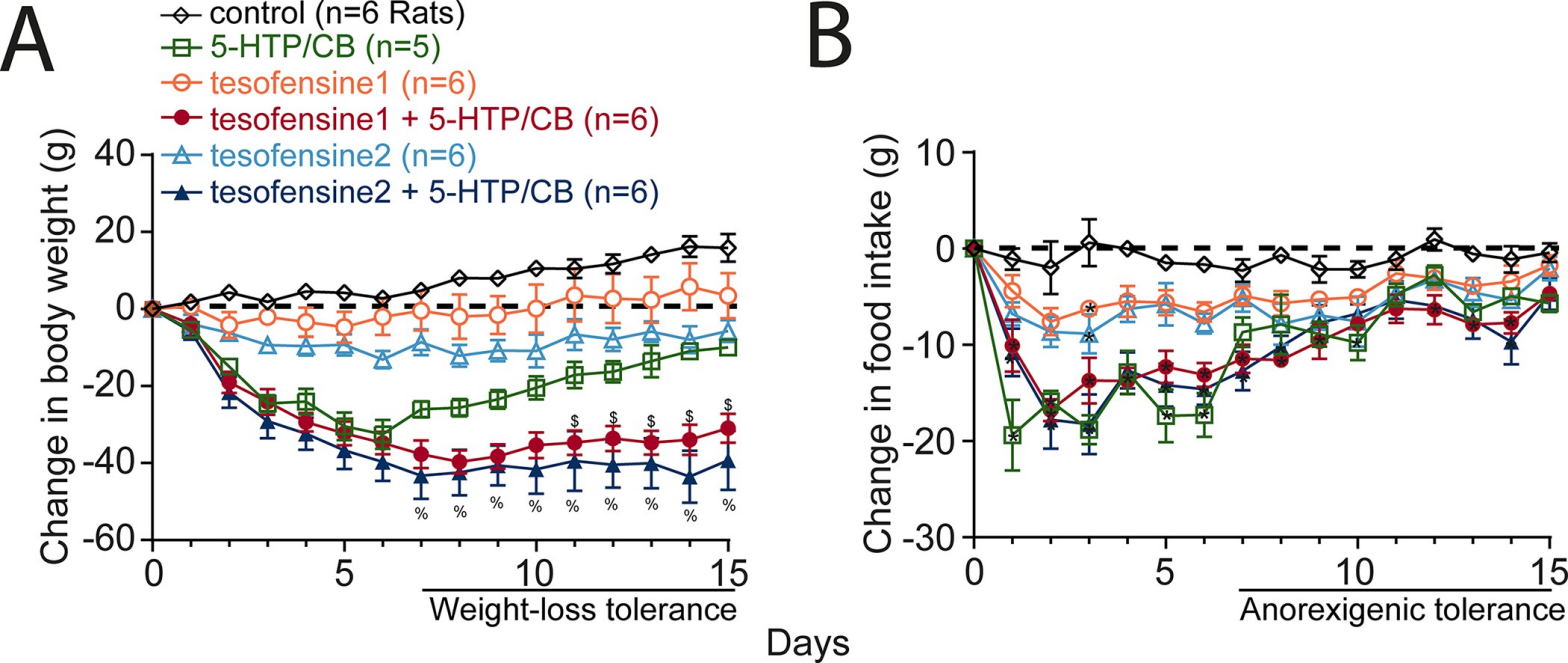
Tesofensine prolongs body weight loss produced by serotonin precursor 5-HTP/CB on the chow control diet in rats. A. Change in body weight (g) relative to pretreatment day 0. Tesofensine1 and tesofensine2 refer to doses of 1 and 2 mg/kg administered subcutaneously, respectively. 5-HTP/CB refers to doses of 31 and 75 mg/kg administered intraperitoneally. Note that the triple combination of tesofensine, 5-HTP, and CB led to significantly greater weight loss than any other group and did not show weight loss tolerance as seen in 5-HTP/CB group (see horizontal line at days 7–15). B. Changes in food intake for the groups shown in panel “A.” Note that 5-HTP/CB, tesofensine1, and 2 + 5-HTP/CB groups exhibited anorexigenic tolerance, as evidenced by their increased food intake from day 7 to 15. Data are presented as mean ± SEM. n = number of rats. Repeated Measures ANOVA (A-B). $ p < 0.05 tesofensine1 compared with tesofensine1 + 5-HTP/CB, and 5-HTP/CB groups. % p < 0.05 tesofensine2 compared to tesofensine2 + 5-HTP/CB, and 5-HTP/CB groups. * p < 0.05 compared with the control group.

In lean animals, we evaluated the weight loss effects and interaction of tesofensine with 5-HTP, another appetite suppressant, by administering each drug alone or in combination. The change in body weight (g) after administration of control (vehicle), tesofensine (1 and 2 mg/kg), 5-HTP (31 mg/kg), and CB (75 mg/kg) is shown in **Fig 8A**. We found a significant difference in body weight among groups [RM ANOVA; main effect doses: F_(5,28)_ = 26.4, *p* < 0.0001; days: F_(14,392)_ = 22.1, *p* < 0.0001; doses per days interaction: F_(70,392)_ = 6.7, *p* < 0.0001]. The control group steadily gained body weight, while tesofensine (1 mg/kg) prevented body weight gain without achieving statistical differences compared to control rats (*p* > 0.3, n.s.). At 2 mg/kg, tesofensine induced modest but significant weight loss from the eighth day of treatment onwards (*p* < 0.05). 5-HTP/CB caused rapid, substantial weight loss for the first six days of treatment but showed gradual weight regain, indicating tolerance. Combining both drugs led to substantial weight loss from 1 to 8 days, which was maintained until the end of treatment. The combination led to greater weight loss than each drug alone (*p* < 0.05). Our results demonstrated that tesofensine reverted the pharmacological weight loss tolerance observed in 5-HTP/CB alone.

Regarding food intake, **Fig 8B** shows the change in food intake that accompanied weight loss. The control group showed no change in food intake (-1 ± 0.2 g). Tesofensine (1 and 2 mg/kg) reduced food intake (-4.7 ± 0.4 g and -6.1 ± 0.5 g, respectively). A more potent anorexigenic effect was induced by 5-HTP/CB alone. 5-HTP/CB alone suppressed food intake for 1–6 days (-16.9 ± 0.9 g) but gradually increased the intake for 7–15 days (-6.6 ± 0.7 g). A similar tolerance to the anorexigenic effects of the combination was also seen. Both combinations, tesofensine1 or 2, + 5-HTP/CB suppressed food intake from 7 to 15 days (-8.1 ± 0.7 g and -7.8 ± 0.8 g, respectively). However, they demonstrated no synergistic effect compared to 5-HTP/CB alone (p > 0.1, n.s.). Hence, the tolerance for anorectic effects was still present in the combination. However, tesofensine entirely reverted the 5-HTP tolerance for weight loss, suggesting a greater effect of tesofensine on energy expenditure than food intake suppression.

### The tesofensine plus 5-HTP/CB combination does not result in synergistic interaction in a sucrose intake suppression assay

**Fig 9:**
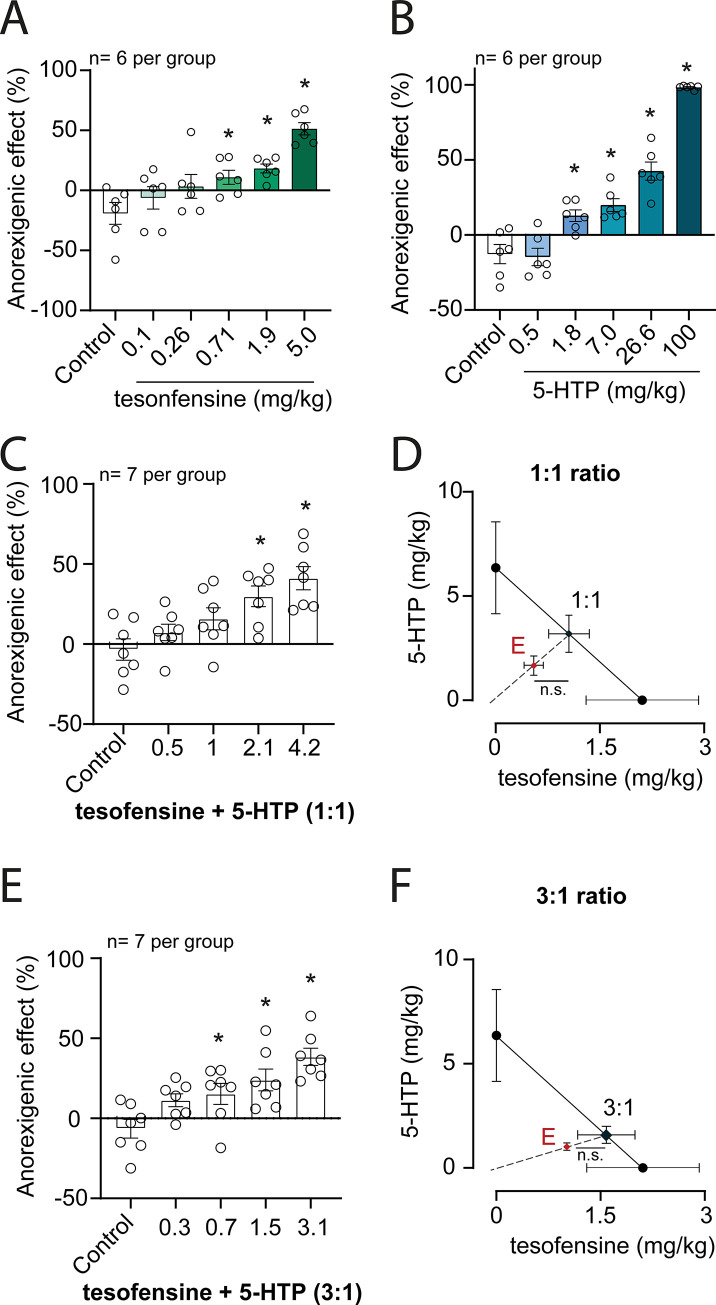
Isobolographic analysis of the interaction between tesofensine and 5-HTP/CB in a sucrose intake assay in rats. The anorexigenic effect was measured as a reduction in sucrose intake (see text for details). Panels A-B depict the dose-dependent effect of tesofensine and 5-HTP/CB, respectively. Data are plotted as mean + SEM, open circles represent individual animals, and * corresponds to a statistically significant difference (p<0.05) with respect to the control group, as determined by one-way ANOVA. C. Depicts the dose-dependent effect of the 1:1 combination of tesofensine and 5-HTP/CB. Data are presented as for panels A and B. D. shows the isobolographic analysis of the 1:1 combination. The oblique line is the isobole corresponding to a purely additive interaction, and black circles represent the ED30 (+ SEM) values of the individual components and the theoretical ED30 value for a purely additive interaction. The red triangle indicates the experimental ED30 of the combination. A comparison of the theoretical and experimental ED30 was performed by the modified Student’s t-test, n.s. indicating a lack of statistically significant difference (p>0.05). E. depicts the dose-dependent effect of 3:1 combination of tesofensine and 5-HTP/CB. Data are presented as for panels A, B, and C. F. shows the isobolographic analysis of the 3:1 combination. Data are presented as in panel D.

The anorexigenic effects of tesofensine, 5-HTP/CB and 1:1 and 3:1 combinations of individual components were assayed by the consumption of sucrose. All treatments produced a significant anorexigenic effect, as determined by a one-way analysis of variance comparing experimental to control groups (**Fig 9**). The interaction between tesofensine and 5-HTP/CB was characterized by isobolographic analysis assuming that the combination comprised equi-effective doses of the individual components. Both tesofensine and 5-HTP/CB produced a dose-dependent reduction in sucrose intake (**Fig 9A** and **9B**). ED30 values were 2.11 ± 0.81 mg/kg for tesofensine and 6.36 ± 2.21 mg/kg for 5-HCP/CB. The combination of tesofensine + 5-HTP/CB 1:1 and 3:1 also induced a dose-dependent anorexigenic effect (**Fig 9C** and **9E**). In both cases, experimental ED30 values were lower than the theoretical values, resulting in an interaction index lower than 1 (**Table 2**). However, differences did not achieve statistical significance. Hence, the interaction between tesofensine and 5-HTP/CB appears additive, not synergistic. The combination of tesofensine and 5-HTP is not more effective than either drug alone in reducing acute sucrose intake.

**Table 2:** Statistical analysis of tesofensine plus 5-HTP interaction.

Tesofensine + 5-HTP/CB	Theoretical ED30 ± SEM	Experimental ED30 ± SEM	Interaction index
1:1	4.24 ± 1.1 mg/kg	2.21 ± 0.1 mg/kg (n.s.)	0.52
3:1	3.17 ± 0.8 mg/kg	2.03 ± 0.3 mg/kg (n.s.)	0.64

ED30 = effective dose 30; SEM = Standard Error of the Mean (SEM), the results were not statistically significant (n.s.) from the theoretical ED30.

**Fig 10:**
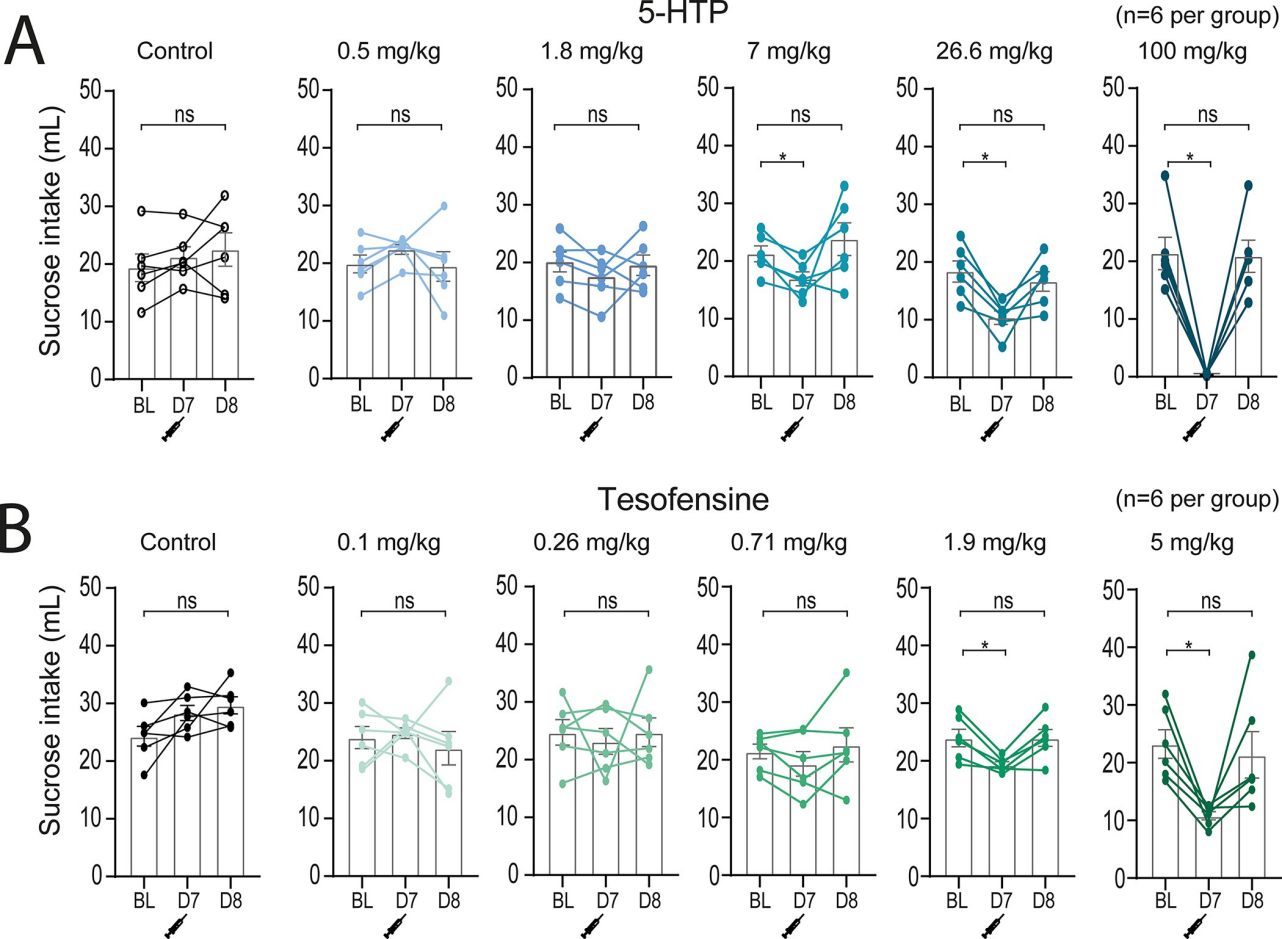
The appetite suppressant effects of tesofensine and 5-HTP/CB is not due to taste aversion. A. The graph plots the average 1-hour sucrose intake during baseline sessions. On day 7 (D7), drugs were administered before giving access to sucrose. A dose-dependent intake suppression can be seen. Finally, on day 8 (D8), intake was observed the day after treatment. 5-HTP/CB suppressed acute sucrose intake on day 7 (D7), but consumption returned to baseline levels on day 8 (D8). Bar plots show acute sucrose intake (1 hour) for control rats (vehicle injection) and treated rats (different doses of 5-HTP/CB). Each dose was tested once in a new set of naïve rats (n = 6 rats per group). Note that 5-HTP/CB induced a dose-dependent decrease in sucrose intake. BL = baseline sucrose intake. B. Same convention as panel “A” but for tesofensine. n = number of rats, open data points represent each rat. One-way ANOVA (A-B).* p<0.05 significantly different from BL.

One probable reason for the appetite-suppressing effect of tesofensine (or 5-HTP) is that it may induce taste aversion. However, our study results do not support this hypothesis. As shown in **Fig 10** the sucrose consumption levels almost returned to baseline after the injection of 5-HTP (**Fig 10A**) or tesofensine (**Fig 10B**) on the next day (day 8). This suggests that taste aversion is unlikely to be the primary mechanism behind the anorexigenic effect of these appetite suppressants.

### Tesofensine does not affect sucrose detection or oromotor palatability responses

**Fig 11:**
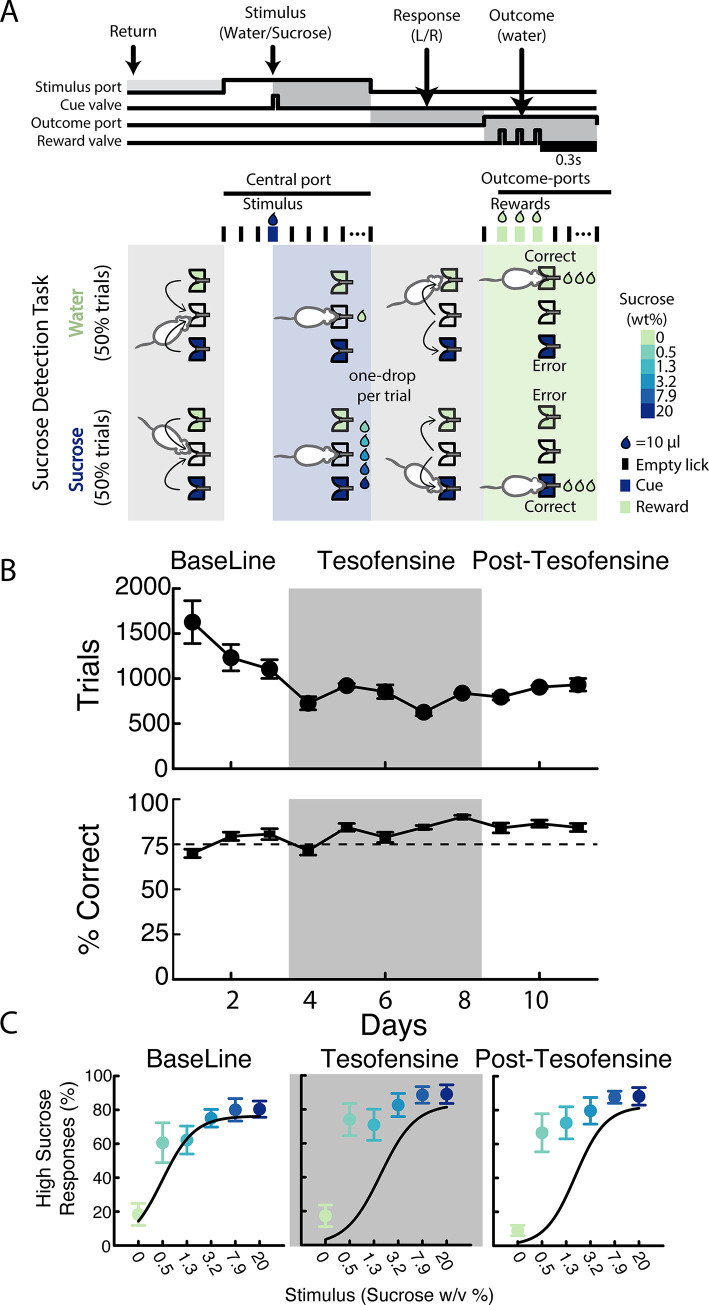
Tesofensine did not affect performance of rats in a sucrose detection task. A. Rats were trained to lick a central spout that dispensed the stimulus a drop of water or solutions of sucrose. To obtain a reward (3 drops of water), rats had to choose between two lateral spouts. B. Upper panel shows the number of trials, and the lower panel the correct performance across the baseline, tesofensine treatment, and post-tesofensine days. There were no significant differences in the percent correct, the trials per session, or the total volume consumed between these periods, except for an overall decrease in the number of trials during the baseline period as the rat re-learned the task. The gray rectangle depicts days of tesofensine administration C. Plots of high sucrose responses as a function of sucrose concentration. The psychometric curves for the sucrose detection task also did not differ significantly between the baseline, tesofensine, and post-tesofensine periods. These findings suggest that tesofensine does not affect performance in the sucrose detection task in rats. The x-axis is scaled logarithmically, and the data is represented as mean ± SEM.

To investigate whether tesofensine impairs sucrose detection or palatability responses, we modified a psychophysical task using a new equipment called the homegustometer [32, 46]. Rats performed the task day and night over multiple consecutive days, which allowed us to characterize the temporal pharmacological effects of tesofensine across days (**Fig 11A**) and within a day (**S3 Fig**). The rats (n = 4) were trained to discriminate between different concentrations of sugar and water using a homegustometer, and their performance was recorded continuously for up to 23 hours daily. At the start of each trial, rats visited the central port and delivered 2–5 dry licks, triggering a taste stimulus, including a single drop of water or one of five sucrose solutions with varying concentrations. The rats discriminated water from sucrose by moving leftward for water and rightward for sucrose solutions (counterbalanced between subjects), with successful discrimination resulting in a reward (three drops of water in lateral outcome sippers). After the rats learned the sucrose detection task, we injected them with 2 mg/kg of tesofensine at ~ 18:00 h for five days. We observed that tesofensine did not impair daily task performance (**Fig 11B**; %Correct). The number of trials and thus total consumption, we observed a temporal increase in the first and second days of baseline that gradually reached an asymptote (**Fig 11B**; Trials and Consumption). Likewise, we observed that psychometric curves during the baseline, tesofensine, and post-tesofensine days were practically identical (**Fig 11C**). However, we also note that under and after tesofensine treatment, the subjects tend to be more sensitive to sucrose, detecting it correctly even at the lowest sucrose concentration (0.5%) (**Fig 11C**). Thus, it is unlikely that the tesofensine anorexigenic properties are because of diminished sweetness detection or changes in their hedonic value or palatability.

### Sucrose detection within a single day

This study investigated the effects of tesofensine administration on the perception of sugar taste in rats over a day. The data were analyzed by dividing the day into quartiles (6-hour periods) to investigate whether tesofensine affected the rats’ perception of the sucrose taste. First, we discuss the performance of the task under control baseline conditions (**S3 Fig**). As expected, given that rats are nocturnal animals, our results showed that they were more likely to perform more trials during the second and third quartiles of the day (Q2 18:00 to 00:00—and Q3 00:00 to 6:00), corresponding to the dark cycle of the day. 30.5% of all trials were completed during the daytime (Q1, Q4), while 69.3% were performed at night (Q2, Q3, **S3 Fig,** panel A; see black percentages). However, the accuracy of the sucrose detection task was not significantly different across the day.

However, rats exhibited a non-significant tendency to decrease performance in Q1 from 12:00 to 18:00 relative to the other quartiles. This poor performance could explain the difficulty of fitting a psychometric curve during Q1 (see **S3 Fig**, right upper panels). The oromotor palatability responses evoked by the delivery of one drop of water or sucrose revealed a gradual increase in the bout size as a function of sucrose, reflecting the hedonically positive reaction that sucrose evokes [33]. In addition, the palatability responses also did not vary across the day (see **S3 Fig**, right lower panels).

The major change observed during the tesofensine treatment was a shift in the distribution of trials completed on each quartile. Specifically, rats performed significantly fewer trials in Q1 and Q2 but compensated for this by performing significantly more in Q3 and Q4. Thus, tesofensine treatment shifted the distribution of trials toward the right. However, the accuracy of the sucrose detection task (i.e., the percent correct trials) was not significantly altered by tesofensine (**S3 Fig**).

Finally, in the post-tesofensine period, rats received subcutaneous injections of saline. Given that the half-life of tesofensine is about 8 days, we continued evaluating the rats’ performance for three more days (**S3 Fig, panel C**). We observed no major change in task performance, or the palatability responses sucrose elicited during this period. Our data suggest that tesofensine in rats did not impair sweetness detection or affect its palatability.

## Discussion

This study found that tesofensine induced greater weight loss in obese rats than in lean Wistar rats. We hypothesized that this was because of tesofensine’s ability to modulate neuronal activity in the LH. Our electrophysiological results showed that tesofensine produced a stronger and larger modulation of LH ensemble activity in obese rats than in lean rats. This suggests that tesofensine may act, in part, by modulating neuronal activity in the LH to reduce food intake and promote weight loss. More importantly, we also found that tesofensine inhibited GABAergic neurons in the LH of Vgat-ChR2 and Vgat-IRES-cre transgenic mice. These neurons promote feeding behavior optogenetically [8, 11], so the inhibition of these neurons by tesofensine may contribute to its appetite-suppressing effects. Besides its effects on the LH, in rats, tesofensine did not produce head weaving stereotypy at therapeutic doses, suggesting that it may be a safer and more tolerable option to treat obesity than other appetite suppressants such as phentermine. It also did not significantly potentiate the acute suppression of sucrose intake induced by 5-HTP, but it prolonged the weight loss induced by 5-HTP, a serotonin precursor and appetite suppressant. This suggests that tesofensine may be a valuable adjunct to serotoninergic agents to treat obesity. Finally, we found that the appetite suppressant effect of tesofensine is not because of the induction of taste aversion. Rats resumed drinking sucrose right after the next treatment day in the isobolographic assay. Further studies using a 23-hour psychophysical sucrose detection task also showed that tesofensine might not affect the perception of sweetness or its palatability responses, even though it is a weight-loss drug. Taken together, our study provides new insights into the effects of tesofensine on weight loss and the underlying neuronal mechanisms. These findings suggest that tesofensine may be a promising new therapeutic agent to treat obesity.

### Tesofensine targets the LH, silencing a subset of GABAergic neurons

Tesofensine is more efficacious in inducing weight loss in obese rats than lean Wistar rats. Our results replicate and confirm the findings observed by Hansen et al., 2013 [3] in Sprague-Dawley rats and [47] in obese Wistar rats, suggesting that this is a robust attribute of tesofensine. They suggested that the greater efficacy was because of the ability of tesofensine to restore lower DA levels in the nucleus accumbens observed in obese rats [3]. Here, we further extend the neuronal correlates to the LH and uncovered for the first time that tesofensine produced a stronger and larger modulation of LH ensemble activity in obese rats than in lean rats. The effects of tesofensine on LH activity were complex. However, tesofensine seems to enhance the recruitment of LH neurons exhibiting activation after drug administration (i.e., see E4 neurons in **Fig 2**). The identity of this cell type is out of the scope of this study, but it is tempting to speculate that most likely includes a large subset of non-GABAergic neurons, perhaps enriched of glutamatergic neurons. We acknowledge that our data cannot rule out the intriguing possibility that a different subset of GABAergic neurons (from those inhibited) could be activated by tesofesnine. This is because activation of GABAergic neurons can trigger oromotor stereotypy [13], similar to that observed with phentermine and tesofensine at high concentrations (see below **Fig 7**). Further studies using Cal-light or TRAP-like techniques should be conducted to confirm the identity of the activated neuronal ensembles recruited by tesofensine [48, 49]. These techniques could capture functional ensembles, enabling more precise identification of the cells that respond to tesofensine and are responsible for its therapeutic anorexigenic effects and stereotypies side effects.

The second larger group of cells that were more strongly modulated by tesofensine in obese than in lean rats was the ensemble of neurons exhibiting a robust inhibition (see E1 in **Fig 2**). Our data in Vgat-IRES-cre mice demonstrate that these neurons correspond to a subset of LH GABAergic neurons (**Fig 3**). We uncovered that tesofensine could silence a subset of optogenetically identified LH GABAergic neurons using optrode recordings. It also impaired their ability to be activated by an open loop optogenetic stimulation (**Fig 3**). Using lean Vgat-ChR2 mice, we found that tesofensine reduces the feeding behavior induced by the optogenetic activation of LH GABAergic neurons (**Fig 4**). Moreover, in Vgat-IRES-cre obese mice, only a higher tesofensine dose could suppress optogenetically induced feeding, suggesting that, during obesity, LH GABAergic neurons seem to be hypersensitized. Thus, these neurons are more resilient to evoked feeding during obesity (**Fig 5**). Conversely, the chemogenetic inhibition of LH GABAergic neurons potentiates the anorexigenic effects of tesofensine (**Fig 6**). Our data is the first to demonstrate that tesofensine directly targets LH feeding circuits, particularly silencing a subset of GABAergic neurons, and activating a still unidentified cell type (perhaps a subset of glutamatergic neurons). It paves the way to uncover better ways to enhance the therapeutic effects of tesofensine and perhaps for other appetite suppressants.

Recently, tesofensine has demonstrated promising results for treating rare human feeding disorders, such as hypothalamic obesity [38]. Hypothalamic obesity symptoms include exacerbated hunger, rapid increase in body weight, and low metabolism. Approximately 50% of craniopharyngioma survivors develop hypothalamic obesity [50]. This type of tumor most often affects the physiological function of the hypothalamus, a part of the brain that regulates appetite and metabolism, thus leading to rapid, intractable weight gain, a condition known as hypothalamic obesity [50]. In particular, the lack of satiety feedback from the hypothalamus has been proposed as a mechanism for hypothalamic obesity [51–53]. Hypothalamic obesity is a challenging condition to treat, as there are currently no approved or effective pharmacological treatments. However, tesofensine is a novel compound with potential in human studies and may be a promising alternative for these patients [38]. Given the ability of tesofensine to modulate the activity of the LH, our preclinical findings agree with the proposal that tesofensine could be a useful treatment for patients with hypothalamic obesity, a rare feeding disorder, as recently demonstrated [38].

### Comparison of tesofensine with other appetite suppressants

#### Tesofensine vs. a dopaminergic agent: phentermine

Tesofensine was initially intended to treat Parkinson’s and Alzheimer’s disease because it increases dopamine efflux. However, in phase II trials, tesofensine was discontinued for lack of potent effects [42, 54, 55]. Unexpectedly, both patients showed significant dose-dependent loss in body weight [56]. Originally, tesofensine was reported to have a greater affinity for dopamine than noradrenaline and serotonin transporters [47]. However, new studies showed that tesofensine (NS2330) has a lower dopaminergic activity and a stronger effect on norepinephrine: NE (IC_50_ 1.7 nM) > DA (6.5) > 5-HT(11), likewise for the major metabolite M1 (NS2360) NE (0.6) > 5-HT(2.0 nM) > DA(3.0) [4, 57]. Accordingly, we found that tesofensine at therapeutic doses did not induce head weaving stereotypy, a classic behavior induced by most, if not all, dopaminergic-acting appetite suppressants [15, 58]. Here, we characterized this behavior in more detail for the first time using a subjective visual human observation of a bottom-view videotape and an automatic DeepLabCut analysis. Our human visual inspection revealed that rats performed tongue movements in the air rather than head weaving, which appears to have no apparent function. Hence, it looks like a head weaving stereotypy from a top view, but the tongue protrusions could be seen from a bottom view videotape. This finding suggests that head weaving in rats may be an indirect indicator of tardive dyskinesia-like behavior, which is characterized by oro-facio-buccal-lingual stereotypic movements [59]. Our DeepLabCut analysis found that forward locomotion was reduced in rats that engaged in head weaving stereotypy for hours, as this stereotypic behavior commonly occurred when rats were standing still on all four limbs (**Fig 7A–7C**). Our data showed that, unlike phentermine, tesofensine at 2 mg/kg induced few, if any, head weaving stereotypy (**Fig 7C**). However, it reduced forward locomotion (**Fig 7A**), suggesting it slightly affected the overall motor map profile relative to control rats that received saline (see **Fig 7E**, see blue dots slightly separated from control black dots). Our findings of a reduction in locomotion are consistent with a previous study [47], although the authors attributed their results to a technical issue. However, we replicated their findings using a more precise method, suggesting that the reduction in locomotion is a genuine phenomenon. Rats treated with tesofensine 2mg/kg commonly transitioned from a sleep-like state to a quiet-awake state. In rare instances, the rats also performed jaw and tongue movements at a lower intensity and for brief periods (see **S8 Video**).

In contrast, only the higher dose of 6 mg/kg induced strong tongue movements in the air, and this stereotypy exhibited some similarities with phentermine. However, we note it was similar but not identical (**Fig 7E**, green vs. yellow dots). This is expected since tesofensine increases striatal DAT occupancy dose-dependently between 18% and 77% in humans [4]. Our results suggest that tesofensine at therapeutic doses does not exhibit strong dopamine activity, as evidenced by the absence of head weaving stereotypies. These findings are also consistent with the low risk of abuse for tesofensine, as it has been reported to be unlikely to be abused recreationally [60].

#### Tesofensine vs. a serotoninergic precursor: 5-HTP

We previously found that the triple combination of phentermine (a dopaminergic compound) plus 5-HTP/carbidopa, a serotoninergic precursor, leads to greater weight loss than each drug alone [25]. Building on this finding, we investigated whether tesofensine could synergistically interact with 5-HTP/CB to prevent weight loss tolerance. We measured body weight and food intake in the homecages of rats while tesofensine alone or in combination with 5-HTP/CB was administered. Two doses of tesofensine (1 and 2 mg/kg) were used. During the first 6 treatment days, our results demonstrated that this combination did not induce more body weight loss than 5-HTP alone; thus, no synergy effect was observed. However, the group treated with 5-HTP/CB exhibited a marked weight loss tolerance in subsequent days and began regaining body weight afterward. Unexpectedly, tesofensine (at both doses) prolonged the weight loss induced by 5-HTP and completely blocked the body weight tolerance (or body weight rebound) over the following days (**Fig 8A**, see days 7–15). This suggests that tesofensine may have two components: one anorexigenic and a second that stimulates energy expenditure, the latter perhaps mediated by its NE component [2, 61]. This is a promising finding suggesting that tesofensine could be an important adjunct for serotoninergic-acting appetite suppressants to prevent the occurrence of body weight tolerance [47, 61].

In this regard, a human study found that subjects who took tesofensine for 24 weeks and then stopped taking it for 12 weeks did not regain all their lost weight [19]. Our results support this finding and extend it by showing that tesofensine can also prevent weight rebound after losing weight with another appetite suppressant.

We next tested the interaction of tesofensine and 5-HTP using a variation of the isobolographic assay, with a 1-hour suppression of sucrose intake as the behavioral response. We observed a tendency for a synergistic effect, as indicated by interaction indices lower than 1 of 0.52 and 0.64 for the 1:1 and 3:1 dose ratio, respectively. However, these differences did not reach statistical significance. Therefore, we concluded that tesofensine and 5-HTP/CB exhibited an additive pharmacological interaction for liquid sucrose intake suppression (**Fig 9**). Unlike the synergistic interaction of phentermine/5-HTP/CB [25], our results again indirectly suggest that tesofensine, at therapeutic doses, has lower dopaminergic activity. This could explain why we did not find a synergistic effect between tesofensine and 5-HTP.

These experiments also revealed that rats recovered sucrose intake the following day after receiving 5-HTP or tesofensine (**Fig 10**). This suggests that taste aversion does not explain the appetite-suppressing effect of these two drugs. Therefore, tesofensine appears to have anorexigenic properties on its own that are not solely dependent on taste aversion.

#### Tesofensine and sweet perception

A human study found that tesofensine increased satiety and decreased cravings for sweet foods after 12 weeks of treatment [19]. However, these effects disappeared after 24 weeks. The reasons for this are unclear [19]. To investigate this further, we used a psychophysical sucrose detection task in rats to determine whether tesofensine affects taste perception. Our data showed that tesofensine did not directly impair the perception of sweetness or its palatability responses (**Fig 11 and S3 Fig**). Although taste responses in rats and humans differ depending on the sweet stimulus, because the homology between sweet receptors in the two species is less than 70% [62], our results suggest that the diminished sweet craving effect observed in humans treated with tesofensine is most likely not due to taste aversion or impairment in sweet perception. Instead, it is likely because of other taste-independent factors, such as post-oral "appetition" signals that mediate food preference via gut-brain nutrient signaling mechanisms [63]. Further studies are needed to clarify this point.

#### Limitations and future directions

Given that tesofensine is a triple reuptake inhibitor that regulates the level of DA, 5-HT, and NE across the entire brain, its effects are expected to be distributed and brain-wide, certainly not restricted to LH or GABAergic neurons. Further studies using high-density recordings of neuropixels need to unveil how distributed tesofensine’s effects are across the brain. In this regard, the balance of neurotransmitters in the brain, specifically norepinephrine (NE), dopamine (DA), and serotonin (5-HT), is a major determinant of the overall weight loss properties of most appetite suppressants [14, 25, 64]. A caveat of our study is that we did not measure the release of these neurotransmitters. Therefore, future studies are warranted to measure NE, DA, and 5-HT simultaneously and map the neurochemical landscape evoked by tesofensine (and other appetite suppressants) using either GRAB sensors with fiber photometry [65, 66] or classic *in vivo* microdialysis with capillary electrophoresis. In addition, it will be relevant to identify the difference either in the distribution or physiological properties of the receptors indirectly targeted by tesofensine in obese versus lean mice. These studies will clarify the neurochemical profile of each appetite suppressant and will guide us in classifying and combining them better.

## Conclusion

Our findings suggest that tesofensine is a promising new therapeutic agent for treating obesity. It modulates neuronal activity in the LH to reduce food intake and promote weight loss. Our data also paves the way for LH GABAergic neurons, among other cell types (perhaps glutamatergic), in the Lateral Hypothalamus to be a potential pharmacological target for developing new appetite suppressants to treat obesity. Additionally, this study found that tesofensine may be a valuable adjunct to serotonergic agents to treat obesity, primarily to prevent body weight rebound.

## Supporting information

S1 Fig The body weight of chow-fed and HFD-fed rats before treatment. Non- significant difference on body weight was present on both groups fed with chow or between both groups fed with HFD. Error bars = standard error of the mean (SEM). n = numbers of rats. Filled and open data points represent rats. n.s. no significant (p>0.05, One-way ANOVA).
(TIF)

S2 Fig Training in the homegustometer of the sucrose discrimination task. A. It shows the performance of four rats in the sucrose discrimination task across sessions, expressed as a percentage of correct responses. The dashed line at 75% correct responses indicates the learning criterion. After five sessions, all subjects were able to distinguish between the different sucrose concentrations (above 75% correct for three consecutive days). B. it depicts the total number of trials as a function of sessions. Individual rats are depicted as grey lines, and the average performance is shown in black. Data are represented as mean ± SEM.
(TIF)

S3 Fig Performance of rats in a sucrose detection task across 23 hours under baseline, tesofensine, and post-tesofensine treatments. A. Baseline performance. The data are presented in 6-hour intervals (divided into four quartiles, Q1–Q4- the moon symbol indicates quartiles during night and sun symbol periods of light) and include the percentage of correct responses and the fraction of trials performed in each quartile across the days. The chance is 50% for the left blue axis. B. Tesofensine treatment (2 mg/kg). The data are presented in the same way as in panel “A.” C. Post-tesofensine treatment. For all panels, the psychometric curves at the right show the percentage of correct choices for detecting sucrose solutions as a function of sucrose concentration. The x-axis of the psychometric curve is scaled logarithmically. Below, the bout size is the time elapsed between the last lick in the central port after stimulus delivery. * Depicts the mean bout size, and each color dot is one single trial. The larger the bout size, the more palatable the drop of solution is to the rat. Our results indicate that sucrose detection and palatability responses were unaffected by tesofensine. The data are represented as mean ± SEM.
(TIF)

S1 Video Quiet-awake-control. (MP4)

S2 Video Sleeping state control. (MP4)

S3 Video Grooming stereotypy control. (MP4)

S4 Video Stereotypy phentermine. (MP4)

S5 Video Tesophensine 2 mg, quiet-awake. (MP4)

S6 Video Control quiet-sleep. (MP4)

S7 Video Tesophensine 2 mg stereotypy. (MP4)

S8 Video Tesophensine 2 mg tongue-stereotypy. (MP4)

S9 Video Tesophensine 6 mg stereotypy. (MP4)

S1 File (ZIP)

S2 File (PDF)

S1 Data (RAR)
