# Supplementary figures and images for: Tesofensine, a novel antiobesity drug, silences GABAergic hypothalamic neurons
Source: PLoS One. 2024 Apr 24;19(4):e0300544. doi: 10.1371/journal.pone.0300544 (PMC11042726; doi:10.1371/journal.pone.0300544)

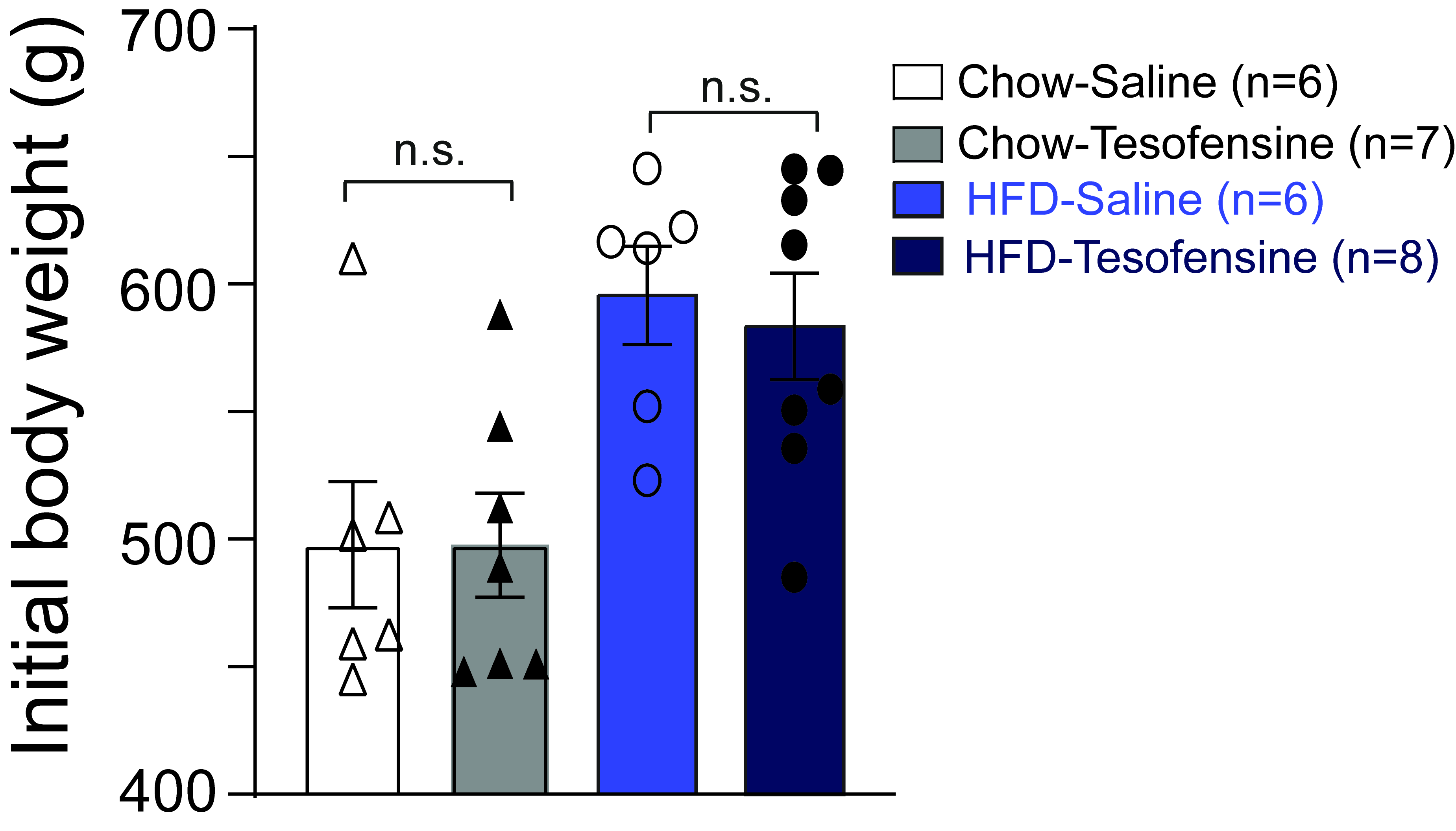

Supplement: S1 Fig — Non- significant difference on body weight was present on both groups fed with chow or between both groups fed with HFD. Error bars = standard error of the mean (SEM). n = numbers of rats. Filled and open data points represent rats. n.s. no significant (p>0.05, One-way ANOVA). (TIF) [file pone.0300544.s001.tif]

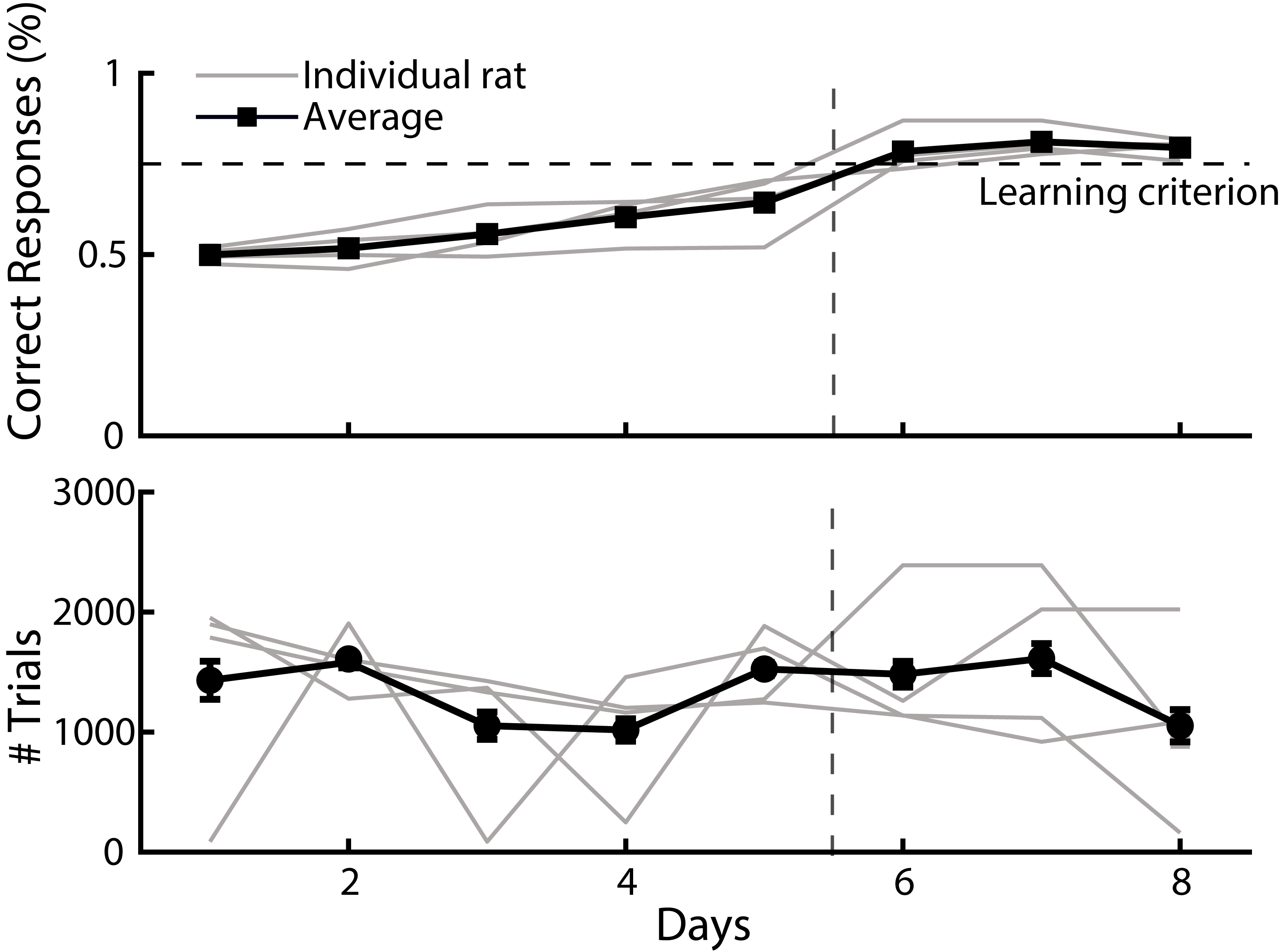

Supplement: S2 Fig — A. It shows the performance of four rats in the sucrose discrimination task across sessions, expressed as a percentage of correct responses. The dashed line at 75% correct responses indicates the learning criterion. After five sessions, all subjects were able to distinguish between the different sucrose concentrations (above 75% correct for three consecutive days). B. it depicts the total number of trials as a function of sessions. Individual rats are depicted as grey lines, and the average performance is shown in black. Data are represented as mean ± SEM. (TIF) [file pone.0300544.s002.tif]

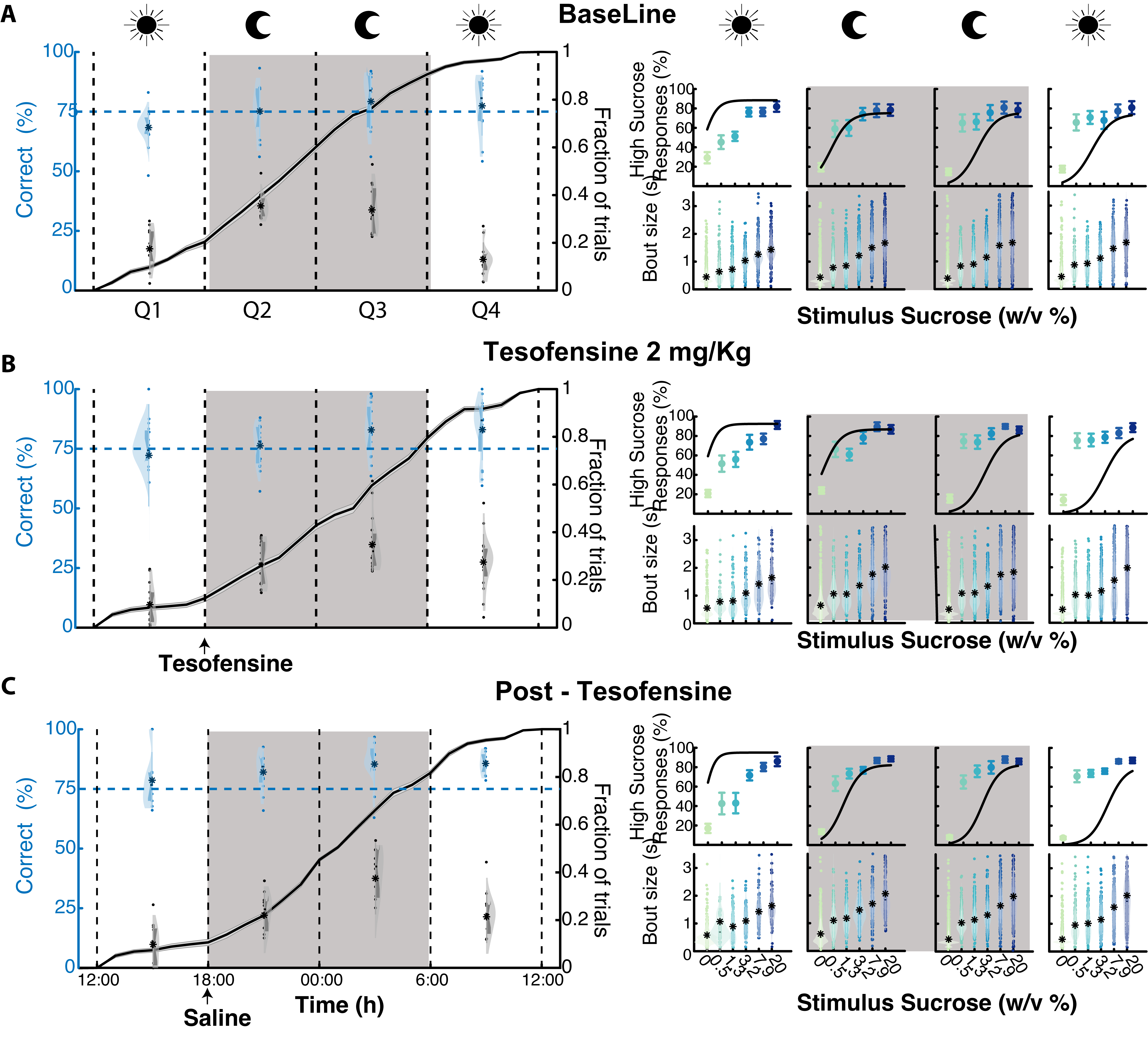

Supplement: S3 Fig — A. Baseline performance. The data are presented in 6-hour intervals (divided into four quartiles, Q1–Q4- the moon symbol indicates quartiles during night and sun symbol periods of light) and include the percentage of correct responses and the fraction of trials performed in each quartile across the days. The chance is 50% for the left blue axis. B. Tesofensine treatment (2 mg/kg). The data are presented in the same way as in panel “A.” C. Post-tesofensine treatment. For all panels, the psychometric curves at the right show the percentage of correct choices for detecting sucrose solutions as a function of sucrose concentration. The x-axis of the psychometric curve is scaled logarithmically. Below, the bout size is the time elapsed between the last lick in the central port after stimulus delivery. * Depicts the mean bout size, and each color dot is one single trial. The larger the bout size, the more palatable the drop of solution is to the rat. Our results indicate that sucrose detection and palatability responses were unaffected by tesofensine. The data are represented as mean ± SEM. (TIF) [file pone.0300544.s003.tif]
