# Supplementary material for: Tesofensine, a novel antiobesity drug, silences GABAergic hypothalamic neurons
Source: PLoS One. 2024 Apr 24;19(4):e0300544. doi: 10.1371/journal.pone.0300544 (PMC11042726; doi:10.1371/journal.pone.0300544)
Supplement: S2 File — (PDF) [file pone.0300544.s014.pdf]

%% Laboratory of Neurobiology of Appetite, Department of Pharmacology,
%CINVESTAV, México

% PhD Ranier Gutierrez, ranier@cinvestav.mx

% Axel Lopez, Claudia I. Perez and Ranier Gutierrez wrote the code

% This MATLAB code is generated according to what is published in the book: "DRUG SYNERGISM and DOSE-EFFECT

% DATA ANALYSIS", Author Ronald J Tallarida

% If you use this code please cite the following manuscript

% Tesofensine, a novel antiobesity drug, silences GABAergic hypothalamic neurons

% Claudia I. Perez, Jorge Luis-Islas, Axel Lopez, Xarenny Diaz, Omar Molina, Benjamin
%Arroyo, Mario G. Moreno, Elvi Gil Lievana, Esmeralda Fonseca, Gilberto Castañeda-
%Hernández, and Ranier Gutierrez

% bioRxiv 2023.08.02.551706; doi: https://doi.org/10.1101/2023.08.02.551706

% Data %%%%%

% To generate the dose-response curve, its necessary to evaluate the effect

% at different doses for DrugA and DrugB. The data that you need to add in this code:

% DosesA: Doses of drug A, [0.5 1.8 7 26.6 100]. Example 0.5, 1.8, 7, 26.6, and 100

% DosesB: Doses of drug B, [0.1 0.26 0.71 1.9 5].

% EffectA: Effective dose of drug A at doses used.

% EffectB: Effective dose of drug B.

% CombinationC: Doses of the combination C, according to effective doses of DrugA and DrugB)

% EffectCombinationC: Effect doses of the combination C.

% ratio: '0.50'; '0.25'; or '0.75' (1:1, 1:3,3:1 ratio, respectively).

```
% ED: '30'; '40'; or '50'. Effective level select and this value would be expected to give
%the desired effect: ED30, ED40, ED50, etc).

% We use the example from Tallarida (Chapter 4), that the ratio is 1:1 and

% ED50. See excel "Raw-data-to-matlab".


DoseA =[1.138 3.793 11.38 37.93];

EffectA =[19.67 40.32 61.91 88.52];

DoseB = [0.8 2.667 7.998 26.66];

EffectB = [19.79 31.4 74.92 92.41];

CombinationC = [1.19 2.38 4.76];

EffectCombinationC = [38.96 65.98 92.04];


ratio = input('Ratio that will be use: ' ); %0.5

ED = input('Effective doses: '); %50

%Ratio that will be use: 1:1

%Effective doses: 50



%% Calculate the effective doses


LogDoseA = log10(DoseA);

LogDoseB = log10(DoseB);

LogCombinationC = log10(CombinationC);

xA = mean(LogDoseA);

xB = mean(LogDoseB);

xC = mean(LogCombinationC);
```

```
yA = mean(EffectA);

yB = mean(EffectB);

yC = mean(EffectCombinationC);



DoseANum = length(DoseA);

DoseBNum = length(DoseB);

CombinationCNum = length(CombinationC);


LogDoseB = LogDoseB';

EffectB = EffectB';

LogDoseA = LogDoseA';

EffectA = EffectA';

LogCombinationC = LogCombinationC';

EffectCombinationC = EffectCombinationC';


%linear regression equations y = mx + b, model = fitlm(LogDoseA,EffectA);

EcA = [LogDoseA ones(size(LogDoseA))];

[m_bA,BintA,RA,A,statsA] = regress(EffectA,EcA);

SlopeA= m_bA(1);

InterceptA = m_bA(2);

R2A =statsA(1);


EcB = [LogDoseB ones(size(LogDoseB))];
```

```
[m_bB,~,~,~,statsB]= regress(EffectB,EcB);

SlopeB= m_bB(1);

InterceptB = m_bB(2);

R2B =statsB(1);


% plotear

% x = min(LogDoseA) : .1 : max(LogDoseA);

% y = m_bB(1)*x +m_bB(2);

% plot(x,y,'m:','linew',6) hold on

% legend({'regresion lineal'},'location','northwest')



EcC = [LogCombinationC ones(size(LogCombinationC))];

[m_bC,BintC,RC,C,statsC] = regress(EffectCombinationC,EcC);

SlopeC= m_bC(1);

InterceptC = m_bC(2);

R2C =statsC(1);



% Standard error of estimate

s2A = statsA(4);

s2B = statsB(4);

s2C = statsC(4);


% Estimated regression line for DrugA, DrugB and CombinationC

lEDA = (ED-InterceptA)/ SlopeA;
```

```
lEDB = (ED-InterceptB)/ SlopeB;

lEDC = (ED-InterceptC)/ SlopeC;


% This is the value of the effective dose of DrugA and DrugB at determinate

% ratio (ED30, ED50, etc)

EDDrugA = 10^lEDA;

EDDrugB = 10^lEDB;

EDC = 10^lEDC; %Zmix


SSregA = sum((LogDoseA-xA).^2);

SSregB = sum((LogDoseB-xB).^2);

SSregC = sum((LogCombinationC-xC).^2);


% The variance of Log Doses (VlogDEA)

VlogDEA = (s2A/SlopeA^2)*((1/DoseANum)+((lEDA-xA)^2)/SSregA);

VlogDEB = (s2B/SlopeB^2)*((1/DoseBNum)+((lEDB-xB)^2)/SSregB);

VlogDEC = (s2C/SlopeC^2)*((1/CombinationCNum)+((lEDC-xC)^2)/SSregC);


% Standard error variances eeLOGDE (SE(logDE50))

EElEDA = sqrt(VlogDEA);

EElEDB = sqrt(VlogDEB);

EElEDC = sqrt(VlogDEC);
```

Example

From Tallarida (2000). Book "DRUG SYNERGISM and DOSE-EFFECT DATA ANALYSIS, Chapter 4 "Calculations for the combination Drug Analysis"

DOSES EFFECT DATA FOR 2 DRUGS

DRUG A

Doses A	Effect A
1.138	19.67
3.793	40.32
11.38	61.91
37.93	88.52

DRUG B

Doses B	Effect B
0.8	19.79
2.667	31.4
7.998	74.92
26.66	92.41

COMBINATION C

Effective level select: example 50 (ED50)

For ratio 3:1, f= 0.25, Ecuation f(ED50DrugA)-(1-f)(ED50DrugB)

For ratio: 1:3, f=0.75

For ratio 1:1, f=0.50

Ratio 1:1

	Doses C	Effect C
ED50/4	1.19	38.96
ED50/2	2.38	65.98
ED50	4.76	92.04

DrugA: Y= 15.64 + 45.21 log(dose), logDrugA= 0.76 ± 0.0234, **ED50(DrugA)= 5.754 ± 0.310**

DrugB: Y= 20.36 + 51.58 log(dose), logDrugB= 0.5746 ± 0.0994, **ED50(DrugB)= 3.755 ± 0.858**

CombinationC: Y= 32.46 + 88.16 log(doseC), log(CombinatioNC)= 0.1989 ± 0.00317, **Zmix= 1.581 ± 0.01151**
